# Aberrant Expression of ACO1 in Vasculatures Parallels Progression of Idiopathic Pulmonary Fibrosis

**DOI:** 10.3389/fphar.2022.890380

**Published:** 2022-07-15

**Authors:** Jutaro Fukumoto, Muling Lin, Mudassir Meraj Banday, Sahebgowda Sidramagowda Patil, Sudarshan Krishnamurthy, Mason Breitzig, Ramani Soundararajan, Lakshmi Galam, Venkata Ramireddy Narala, Colleen Johns, Kapilkumar Patel, John Dunning, Richard F. Lockey, Nirmal S. Sharma, Narasaiah Kolliputi

**Affiliations:** ^1^ Division of Allergy and Immunology, Department of Internal Medicine, University of South Florida, Tampa, FL, United States; ^2^ Department of Molecular Medicine, University of South Florida, Tampa, FL, United States; ^3^ Pulmonary, Critical Care & Sleep Medicine, Morsani College of Medicine, University of South Florida, Tampa, FL, United States; ^4^ Department of Zoology, Yogi Vemana University, Kadapa, India; ^5^ Division of Cardiothoracic Surgery, Department of Surgery, University of South Florida, Tampa, FL, United States; ^6^ Advanced Lung Diseases & Lung Transplantation, Department of Internal Medicine, Morsani College of Medicine, University of South Florida, Tampa, FL, United States; ^7^ Pulmonary and Critical Care Medicine, Brigham and Women’s Hospital, Harvard Medical School, Boston, MA, United States

**Keywords:** lung injury, ACO1, IRP1, IPF, angiogenesis

## Abstract

**Rationale:** Idiopathic pulmonary fibrosis (IPF) is characterized by mitochondrial dysfunction. However, details about the non-mitochondrial enzymes that sustain the proliferative nature of IPF are unclear. Aconitases are a family of enzymes that sustain metabolism inside and outside mitochondria. It is hypothesized that aconitase 1 (ACO1) plays an important role in the pathogenesis of IPF given that ACO1 represents an important metabolic hub in the cytoplasm.

**Objectives:** To determine if ACO1 expression in IPF lungs shows specific patterns that may be important in the pathogenesis of IPF. To determine the similarities and differences in ACO1 expression in IPF, bleomycin-treated, and aging lungs.

**Methods:** ACO1 expression in IPF lungs were characterized and compared to non-IPF controls by western blotting, immunostaining, and enzymatic activity assay. ACO1-expressing cell types were identified by multicolor immunostaining. Using similar methods, the expression profiles of ACO1 in IPF lungs versus bleomycin-treated and aged mice were investigated.

**Measurements and main results:** Lower lobes of IPF lungs, unlike non-IPF controls, exhibit significantly high levels of ACO1. Most of the signals colocalize with von Willebrand factor (vWF), a lineage marker for vascular endothelial cells. Bleomycin-treated lungs also show high ACO1 expressions. However, most of the signals colocalize with E-cadherin and/or prosurfactant protein C, representative epithelial cell markers, in remodeled areas.

**Conclusions:** A characteristic ACO1 expression profile observed in IPF vasculatures may be a promising diagnostic target. It also may give clues as to how *de novo* angiogenesis contributes to the irreversible nature of IPF.

## Introduction

One of the pathognomonic histological features that distinguish IPF from other fibrotic lung diseases is aberrant cell proliferation and metabolism; targeting them is a reasonable strategy to develop new therapies (Vuorinen et al., 2008; Zhao et al., 2017). To date, signaling molecules such as transforming growth factor-beta1 (TGF-β1) and interleukin (IL)-6 have been linked to the profibrotic properties of fibroblasts and lung epithelial cells (Moodley et al., 2003; Fernandez and Eickelberg, 2012). Meanwhile, metabolism is not well studied, especially its relevance to cell proliferation. Identification of dysregulated metabolites or enzymes at fibrotic sites could potentially be of diagnostic and/or therapeutic value given that IPF features an uncontrolled proliferation of metaplastic epithelial cells and fibroblasts (Selman and Pardo, 2006; Xia et al., 2008).

Aconitases are a family of enzymes that catalyze isomerization between citrate and isocitrate in the tricarboxylic acid cycle (TCA cycle). Mammalian cells possess two distinct isoforms of aconitase encoded by different genes: Aconitase 1 [ACO1; cytosolic aconitase (c-aconitase)] encoded by *ACO1* and Aconitase 2 [ACO2; mitochondrial aconitase (m-aconitase)] encoded by *ACO2*. In addition to the enzymatic roles in the cytoplasm, ACO1 also is known to bind to mRNAs of specific genes and maintain iron homeostasis (Anderson et al., 2012). Emerging evidence suggests that mitochondrial dysfunction is contributory to the pathogenesis of IPF (Bueno et al., 2015; Rangarajan et al., 2017; Schuliga et al., 2018; Caporarello et al., 2019; Tsitoura et al., 2019). However, details about the non-mitochondrial enzymes that help to sustain the proliferative nature of IPF are unclear. A 2017 study revealed that cis-aconitate, an intermediate metabolite generated through an aconitase-catalyzed enzymatic reaction, is significantly elevated in IPF lungs (Zhao et al., 2017). However, it is unclear which aconitase isoform(s) causes the accumulation of cis-aconitate in IPF. Furthermore, the mechanisms involved in cis-aconitate accumulation and its impact on fibrogenesis are not yet delineated. It is hypothesized that ACO1 (cytoplasm-localized aconitase isoform) plays a critical role in the IPF metabolome, more specifically, in the proliferation of certain cells and in the pathogenesis of IPF given that enzymatic functions in mitochondria are impaired in IPF (Zhao et al., 2017).

## Materials and Methods

Please see the online supplement for details of materials and methods.

### Cell Culture

The following cell lines and cell types were propagated using established methods or according to the manufacturer’s instructions. A549, H441 cell lines, Human Bronchial Epithelial Cells (ATCC, VA), Human Lung Microvascular Endothelial Cells (Lonza, MD), T7 cells (mouse type II alveolar epithelial cell line; Sigma-Aldrich, MO), C22 cells (mouse club cell line; Sigma-Aldrich), and primary lung fibroblasts.

### Isolation of Mouse Lung Fibroblasts

Primary fibroblasts were isolated from the lungs of WT mice at 7–9 weeks of age using enzymatic digestion based on published techniques with some modifications (Seluanov et al., 2010).

### Plasmids and Transfection

pCMV6-Entry and pCMV6-Entry-ACO1 were purchased (OriGene Technologies, Rockville, MD). pcDNA3.1/V5-His-TOPO and pcDNA3.1/V5-His-TOPO-pro-SPC^WT^ are a gift from Dr. Pascale Fanen (Mondor Institute of Biomedical Research, Paris, France). Transfection of the plasmids was performed using Lipofectamine 2000 (Invitrogen, Life Technologies, CA) as described previously (Fukumoto et al., 2019).

### Murine Lung Fibrosis Model

Age (7–9 weeks) and sex-matched C57BL/6J mice were utilized for the bleomycin fibrosis model. 50 μl of sterile phosphate-buffered saline (PBS) (control group) or bleomycin (1.5 U/kg; treatment group) dissolved in 50 μl of PBS was intratracheally administered. Lung samples were collected as described previously using an institutionally approved protocol (Fukumoto et al., 2013).

### Aconitase Activity Assay

The activity of aconitase in the lung homogenate was measured using a commercially available enzymatic assay kit (Sigma Aldrich, St louis, MO).

### Human Lung Samples

This study involving human tissues adheres to the Declaration of Helsinki and has received approval from the University of South Florida Institutional Review Board (IRB) charged with oversight of human studies (IRB protocol Pro00032158). All human explant lung tissues were obtained from subjects with end-stage lung disease undergoing lung transplantation at Tampa General Hospital, a University of South Florida Morsani College of Medicine affiliated institution, Tampa, Florida (Table 1). All patients gave informed consent prior to study enrollment.

**TABLE 1:** Demographic characteristics, diagnosis and pulmonary function tests of the patients.

Pt No	Age	Gender	Race	Dx	FVC	FEV1
1	67	M	White	COPD	3.18 (68)	1.11 (31)
2	41	M	White	PAH	3.73 (57)	2.66 (52)
3	30	M	White	CF	2.17 (44)	0.97 (24)
4	46	M	Hispanic	DM-ILD	3.60 (59)	2.62 (63)
5	55	M	White	Chronic HP	1.66 (41)	1.37 (43)
6	58	F	Black	IPF	1.11 (47)	0.76 (40)
7	69	M	White	IPF	2.08 (52)	1.92 (62)
8	47	M	White	IPF	3.57 (65)	2.74 (63)
9	61	M	White	IPF	1.33 (34)	1.01 (32)
10	67	M	White	IPF	2.07 (47)	1.62 (47)
11	61	F	Black	MCTD	1.64 (69)	1.02 (53)
12	61	M	White	Chronic HP	1.30 (27)	1.16 (38)
13	65	M	White	IPF	1.54 (33)	1.32 (36)
14	67	M	White	IPF	3.10 (66)	2.10 (60)
15	65	F	White	IPF	0.85 (31)	0.75 (35)
16	52	M	White	IPF	1.47 (34)	1.20 (34)

Dx, diagnosis; COPD, chronic obstructive pulmonary disease; PAH, pulmonary arterial hypertension; CF, cystic fibrosis; DM-ILD, dermatomyositis-associated interstitial lung disease; HP, hypersensitivity pneumonitis; MCTD, mixed connective tissue disease; FVC, forced vital capacity; FEV1, forced expiratory volume in one second. The unit for FVC and FEV1 is litter. For each of the figures in the parenthesis of FVC, the column is the percentage of the predicted FVC, value. Each of the figures in the parenthesis of the FEV1 column is the percentage of the predicted FEV1 value.

### Western Blotting

Protein lysates were prepared from human lung tissues, cell cultures, and mouse lungs based on established protocols as described in the online supplement.

### Immunohistochemical Staining and Protein Quantification on Lung Tissue Sections

Immunohistochemical (IHC) staining was done based on standardized protocols (Fukumoto et al., 2016). After IHC staining, quantification of the gross signal intensity for the targeted protein was performed using ImageJ ver 2.0.0/FIJI (Rizzardi et al., 2012). Details of the antibodies used in this study are provided in the online supplementary document.

### Prediction of Nuclear Localization Signal Sequences

The domain diagram was prepared with Chimera, a visualization system for exploratory research and analysis (Pettersen et al., 2004) using the structure of human ACO1/IRP1 available at the protein data bank [Protein Data Bank (PDB)-ID: 2B3X] (Dupuy et al., 2006). The prediction of nuclear localization signal sequences (NLS) was performed using the NLS mapper (http://nls-mapper.iab.keio.ac.j*p*/cgi-bin/NLS_Mapper_form.cgi) (Kosugi et al., 2009). The predicted NLS for ACO1 that shows the highest score was used to make 2D and 3D domain structures for ACO1 (Supplementary Figure SE5).

### Statistical Analysis

Student’s t-test and Mann–Whitney U test were used, respectively for parametric and non-parametric comparisons. The *p*-value was adjusted using Bonferroni’s method when multiple comparisons were performed on data comprised of more than two groups. GraphPad Prism Version 7.0 (San Diego, CA) was used for analysis.

## Results

### The Expression of Aconitase 1 is Significantly Elevated in the Lower Lobes of IPF Lungs Compared to the Upper Lobes

**FIGURE 1:**
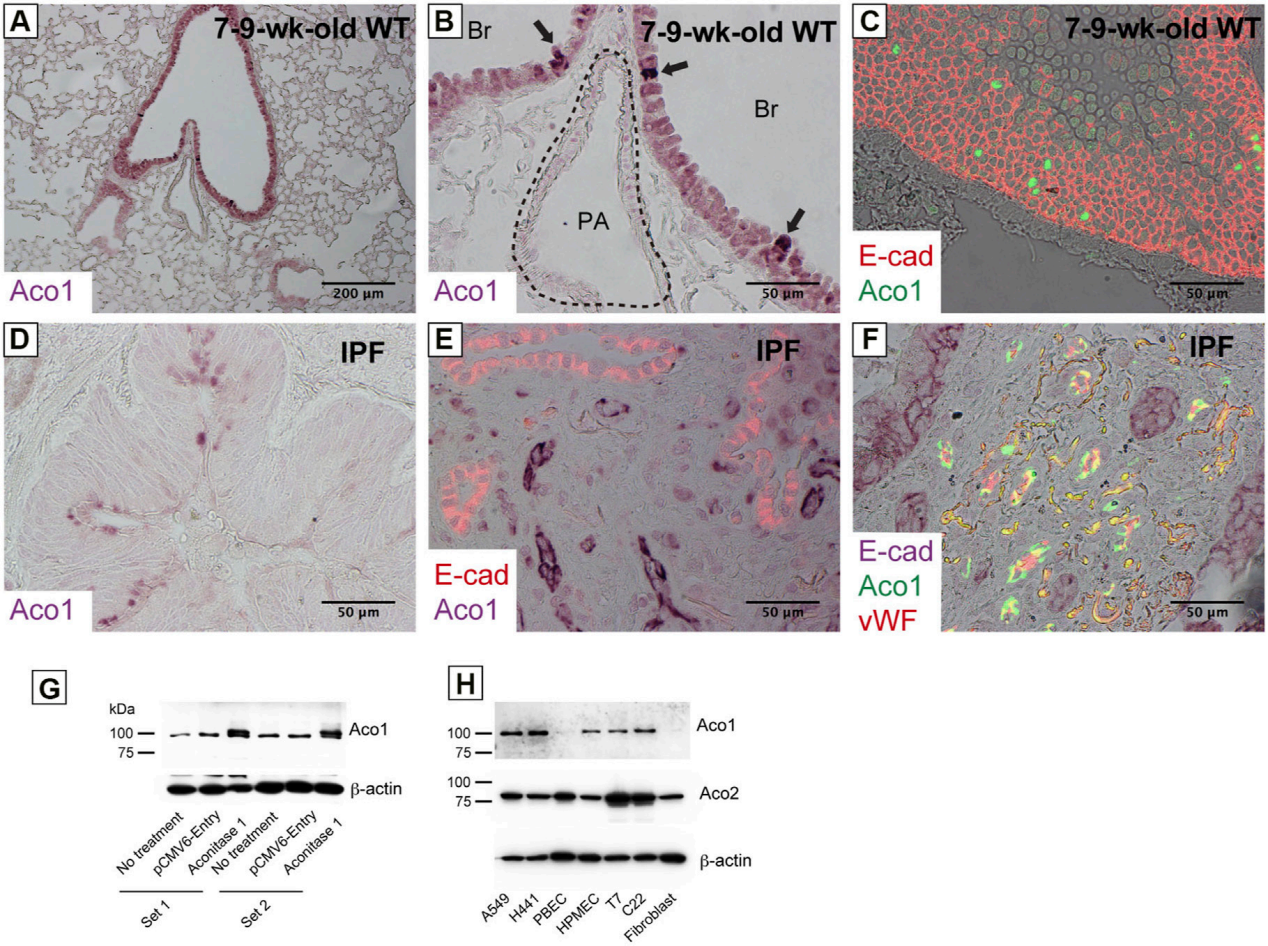
Various cell types in the lung express aconitase 1 in a different subcellular localization pattern **(A–C)** Paraffin-embedded lung sections from wild-type mice aged 7–9 weeks were immunohistochemically labeled for i) Aconitase 1 (ACO1: **(A,B)** or ii) E-cad and ACO1 **(C) (D–F)** Paraffin-embedded lung sections from patients with idiopathic pulmonary fibrosis (IPF) were subjected to immunohistochemical labeling for i) ACO1 **(D)**, ii) E-cad and ACO1 **(E)** or iii) E-cad, ACO1 and vWF **(F) (G)** Specificity of the ACO1 antibody used in this article (cat# PA5-41753, Thermo Fisher Scientific) was evaluated by western blot analysis using whole cell lysates from non-treated H441 cells and H441 cells transfected with either ACO1-overexpression or control plasmids. Equal amounts of protein (7.5 μg) were loaded per lane **(H)** The expressions of ACO1 in different cell types were examined by western blotting. PBEC (Human Pulmonary Bronchial Epithelial Cells); HMVEC (Human Lung Microvascular Endothelial Cells); T7 (mouse immortalized type II alveolar epithelial cell line); C22 (mouse immortalized club cell line); Fibroblast (primary mouse lung fibroblasts). Equal amounts of protein (7.5 μg) were loaded per lane. Data from one representative experiment of two or more independent experiments are shown.

**FIGURE 2:**
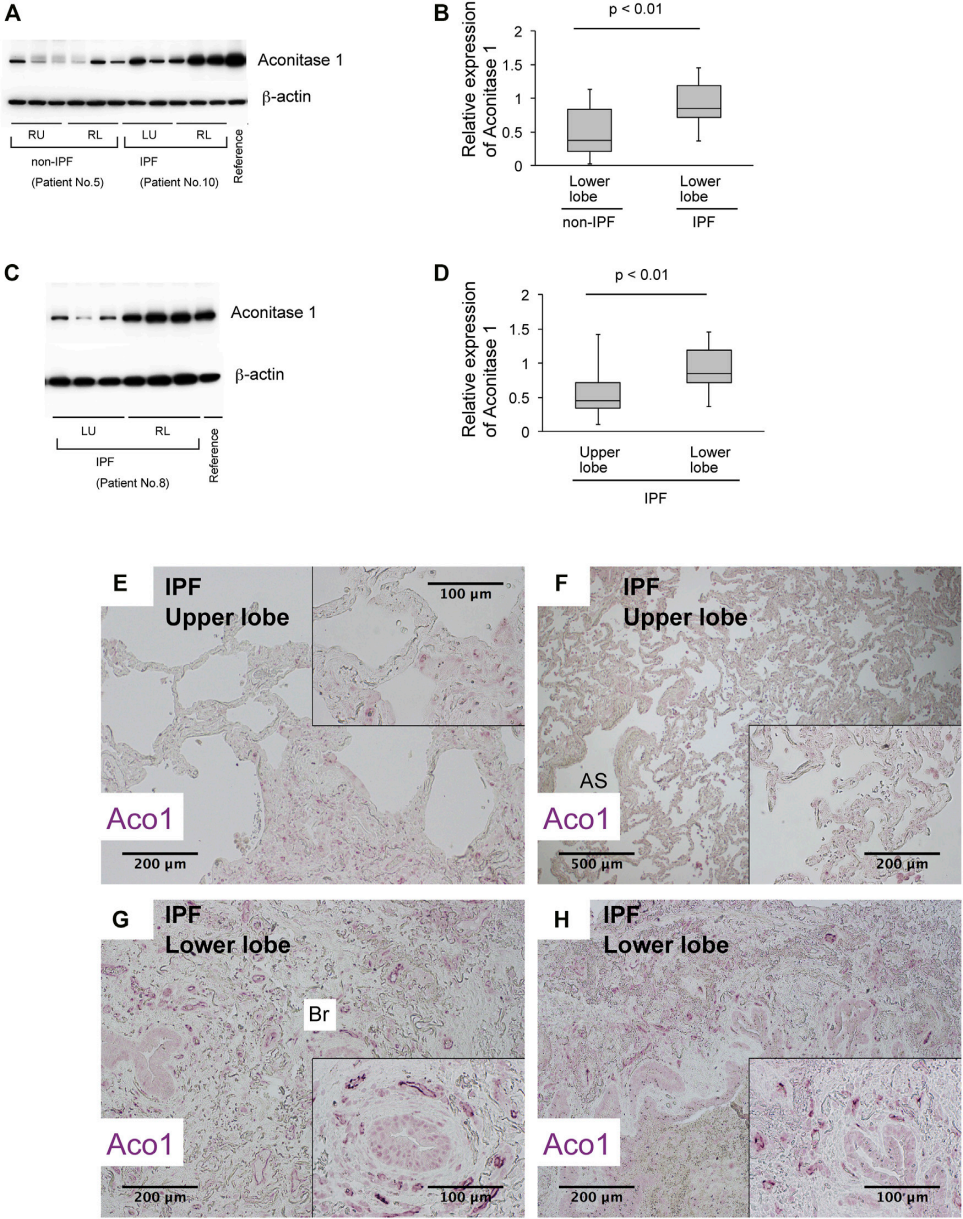
The expression of aconitase 1 is significantly high in the lower lobes of IPF lungs compared to the upper lobes **(A,C)** Western blot analysis for Aconitase 1 (ACO1) was performed using whole lysates prepared from the native lungs of lung transplant recipients with various lung diseases. Each lane represents an individual sample from different sampling sites (please see Figure 3A and online supplement for sampling method). Equal amounts of protein (7.5 μg) were loaded per lane. A common reference sample from Patient No.3 was loaded at the last lane for each blot to enable the quantitative comparison between different blots. RU (right upper lobe); RL (right lower lobe); LU (left upper lobe); LL (left lower lobe) **(B,D)** Quantitative analysis of ACO1 expression based on the results of western blotting. **(B)** Lower lobe samples from Non-IPF (*n* = 18) vs. lower lobe samples from IPF group (*n* = 18). **(D)** Upper lobe samples from IPF (*n* = 15) vs. lower lobe samples from IPF group (*n* = 21) **(E–H)** Paraffin-embedded lung sections from patients with IPF were subjected to immunohistochemical labeling for ACO1. The insets at the corner of each photomicrograph are magnified views of each image. A *p*-value of 0.05 or lower was considered to be statistically significant. Data from one representative experiment of two or more independent experiments are shown.

Previous studies show increased cis-aconitate in IPF (Zhao et al., 2017). It is hypothesized that the accumulation of cis-aconitate in IPF is mediated by a representative non-mitochondrial aconitase isoform, ACO1 (cytoplasm-localized aconitase isoform), since mitochondrial functions are impaired in IPF (Bueno et al., 2015; Rangarajan et al., 2017; Schuliga et al., 2018; Caporarello et al., 2019; Tsitoura et al., 2019). The following were investigated: i) the expression levels of ACO1 in various lung-derived cell types and ii) the expression patterns of ACO1 in human and mouse lungs. Results demonstrate that all cell types tested, except bronchial epithelial cells and lung fibroblasts, express high levels of ACO1 (Figure 1H). Lung sections from untreated wild-type mice and from humans with different disease states were immunohistochemically labeled for ACO1 to further characterize the cell-type-specific expression profiles of ACO1 (Figures 1A–F; Supplementary Figure SE2). The expression of ACO1 in mouse lung epithelial cells was heterogeneous, with intense immunofluorescent signals along the bronchiolar epithelium (Figures 1A–C). In human lungs, ACO1 signals were detected in von Willebrand factor (vWF)-positive vascular endothelial cells (VECs) (Figure 1F; Supplementary Figure SE2E, SE2F) and bronchiolar epithelium (Figure 1D). In addition, ACO1 signals were detected in certain cell types in airspace (Supplementary Figure SE2A, SE2D). The expression of ACO1 in IPF vs. other chronic lung diseases was investigated by western blotting since IPF displays abnormal remodeling in parenchyma as well as in the interstitium. The results showed no significant difference in ACO1 expression between the whole lung samples from IPF and non-IPF patients (data not shown). However, the ACO1 expression in the lower lobes of IPF lungs was significantly higher compared to the control (Figure 2B) or upper lobes of the IPF group (Figure 2D). Such aberrant expressions of ACO1 in the lower lobes of IPF lungs parallel clinical as well as histological manifestations of IPF progression (Figure 2E-H; CT images in Supplementary Figure SE6).

### High Levels of Aconitase 1 Expression in IPF Lungs Reflect Proliferation and Accumulation of Vascular Endothelial Cells

Lung sections from IPF and non-IPF were immunohistochemically labeled for ACO1 and lineage markers, vWF and E-cadherin (E-cad), to identify the cell types that highly express ACO1 (Supplementary Figure SE2). The results show that ACO1-positive cells are encountered in the densely distributed small tubular structures in the lower lobe of IPF lungs (Figures 2 G, H; Supplementary Figure SE2I, SE2L) are mostly positive for vWF (Figure 1F; Supplementary Figure SE2F). Lung sections were labeled for i) ACO1, Ki67, and E-cad (Supplementary Figure SE3A-F) or ii) vWF, Ki67, and E-cad (Figure E3G and E3H) to determine if an abundance of ACO1-positive VECs in IPF is attributed, at least in part, to their active proliferation. These results show that vWF- and ACO1-positive cells in the interstitium of severely fibrotic areas frequently express Ki67 (Supplementary Figure SE3) and indicate that the immunohistochemically detectable ACO1 signals reflect actively proliferating VECs.

### The Total Enzymatic Activity of Aconitase Enzymes Positively Correlates With the Expression of Aconitase 1 in IPF Lungs

**FIGURE 3:**
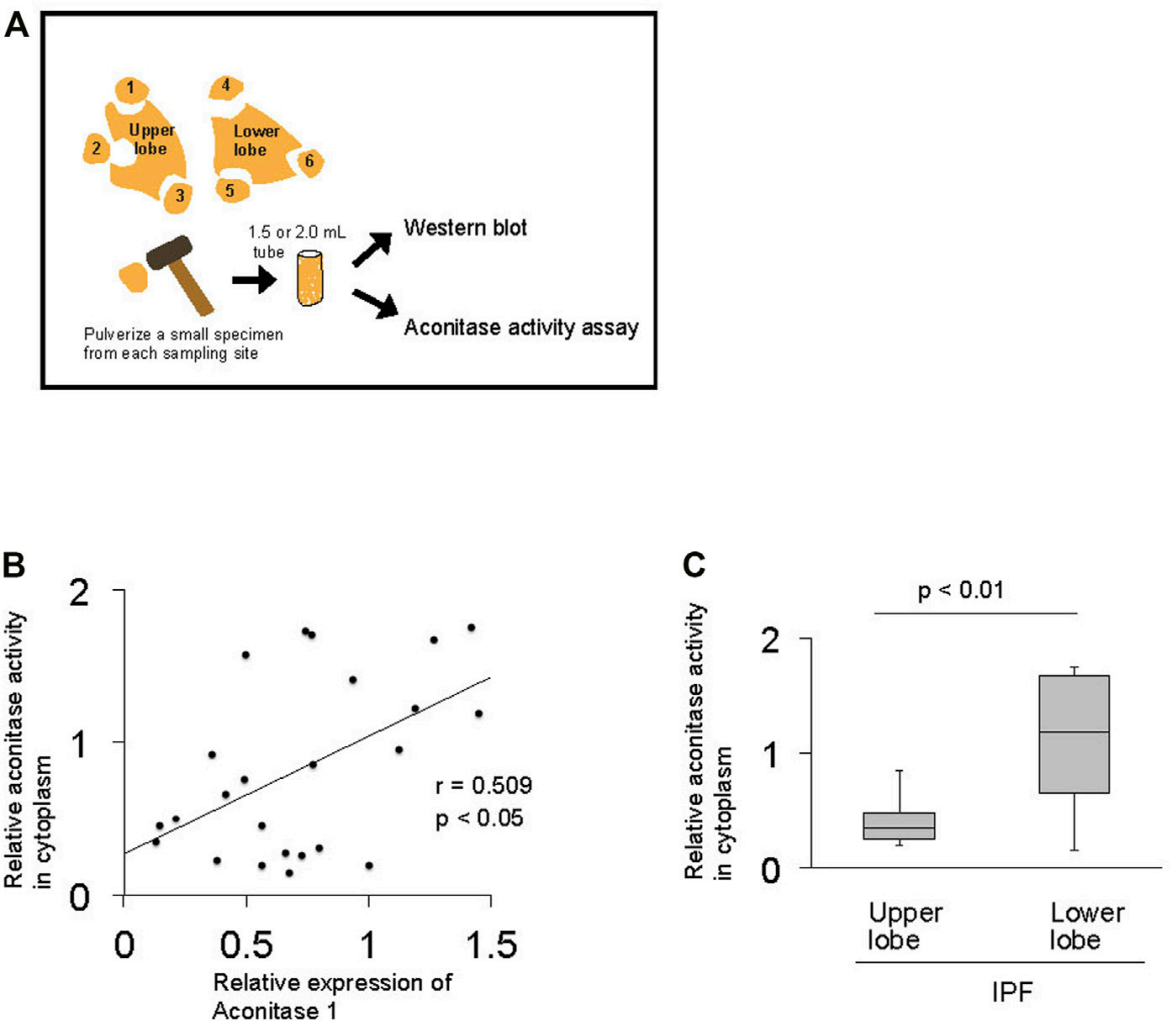
The total enzymatic activity of aconitase enzymes positively correlates with the expression of aconitase 1 in IPF lungs **(A)** Method applied to investigate the correlation between the total enzymatic activity of aconitase enzymes and the expression of aconitase 1 (ACO1) **(B)** Scatterplot of correlation between the expression of ACO1 and total aconitase activity in lung samples (*n* = 24) collected from IPF patients **(C)** Quantitative analysis of aconitase activity: upper lobe samples from IPF (*n* = 9) vs. lower lobe samples from IPF group (*n* = 15). The correlation coefficient (r) and *p*-value (*p*) for each scatterplot are shown. A *p*-value of 0.05 or lower was considered to be statistically significant. Data from one representative experiment of two or more independent experiments are shown.

Previous studies demonstrate that ACO1 assumes either an enzymatic or RNA-binding form based on extracellular factors such as iron and oxygen levels (Meyron-Holtz et al., 2004). The relationship between aconitase activity and the expression of ACO1 was investigated to delineate whether the enzymatic or RNA-binding forms of ACO1 are associated with their increased expression in fibrotic areas of IPF lungs (Figure 3A). The results show that the total aconitase activity and the expression of ACO1 are positively correlated in IPF lungs (Figure 3B, C). Such a positive correlation was not observed between the total aconitase activity and ACO2 expression in IPF (data not shown).

### The Expression of Aconitase 1 and the Total Aconitase Activity Negatively Correlate With the Pro-SPC Expression in IPF

**FIGURE 4:**
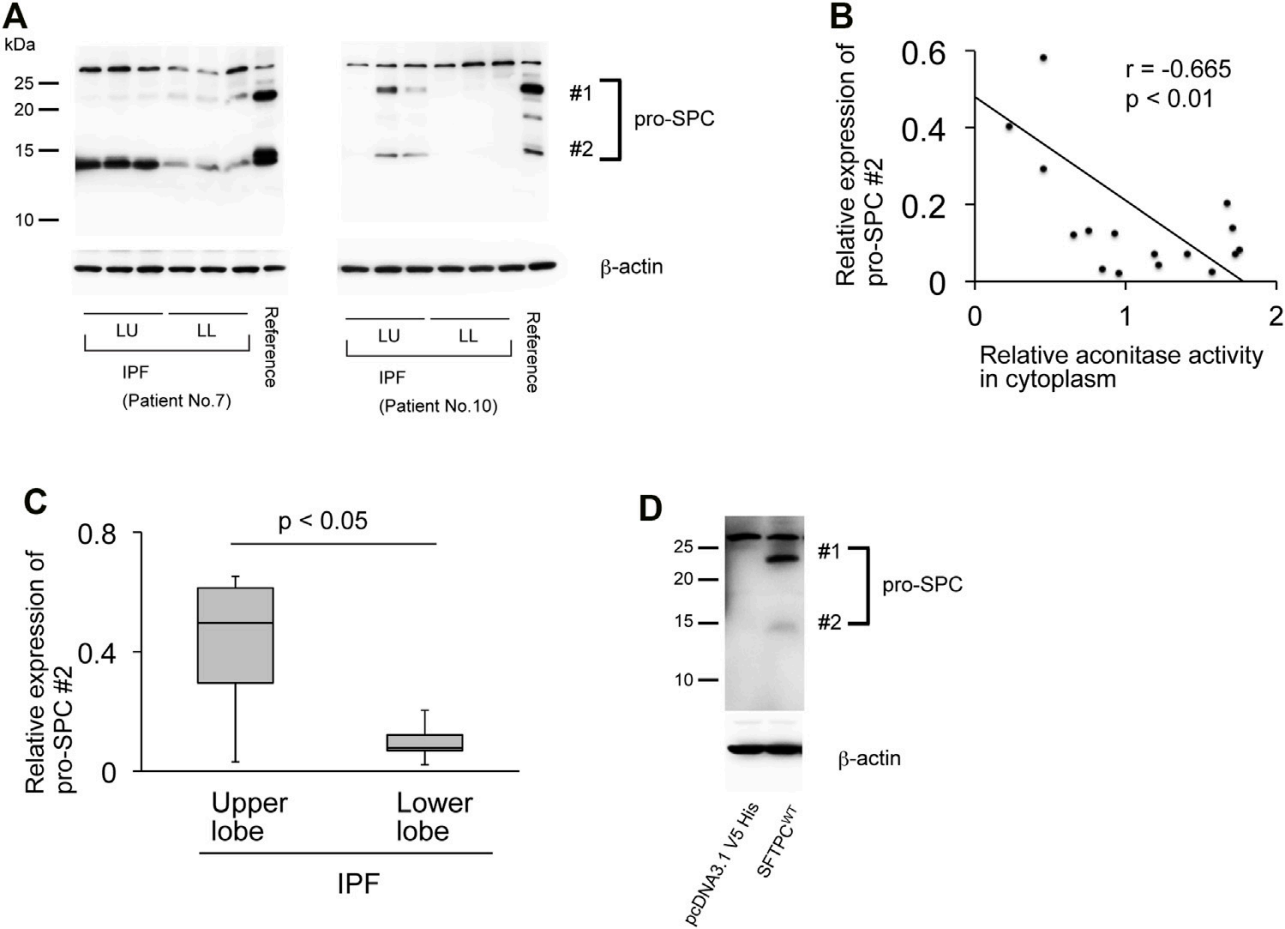
The expression of aconitase 1 and the total aconitase activity negatively correlate with the pro-SPC expression in IPF **(A)** Western blot analysis of pro-SPC using whole lysates prepared from the native lungs of the recipients of lung transplantation performed against IPF. Each lane represents an individual sample collected from different sampling sites (please see Figure 3A for the sampling method). Equal amounts of protein (7.5 μg) were loaded per lane. A common reference sample from Patient No.3 was loaded at the last lane for each blot to enable the quantitative comparison between different blots. LU (left upper lobe); LL (left lower lobe) **(B)** Scatterplot of correlation between aconitase activity and the expression of 12-kD processing intermediate of pro-SPC (#2 in **(A)** in lung samples collected from IPF patients (*n* = 18). The correlation coefficient (r) and *p*-value (*p*) are shown. *p < 0.05. A *p*-value of 0.05 or lower was considered to be statistically significant **(C)** Relative expressions of 12-kDa processing intermediates of pro-SPC (#2 in **(A)** [upper lobe samples from IPF (*n* = 6) vs. lower lobe samples from IPF (*n* = 12)] were shown as a box-whisker plot. Each box shows the upper and lower quartile with the central bar representing the median and the whiskers showing the minimum and maximum **(D)** Specificity of the pro-SPC antibody used in this article (cat# AB3786, SigmaMillipore) was evaluated by Western blot analysis using whole cell lysates from A549 cells transfected with either pro-SPC-overexpression or control plasmids. Equal amounts of protein (7.5 μg) were loaded per lane. Data from one representative experiment of two or more independent experiments are shown.

Pro-fibrotic phenoconversion of type II AECs is implicated in IPF pathogenesis (Selman and Pardo, 2006; Tanjore et al., 2015; Goldmann et al., 2018). The expression of prosurfactant protein C (pro-SPC) is known to predict the functional integrity of type II AECs (Marmai et al., 2011). Therefore, the pro-SPC expression in its relation to ACO1 in fibrotic areas of IPF lungs was investigated. The pro-SPC antibody used in western blotting (cat# AB3786; MilliporeSigma) detected two pro-SPC-specific bands, 21-kDa and 12-kDa (Figure 4D) (Borok et al., 2020). It is surmised that in light of the previously established maturation process of pro-SPC, the 21-kDa and 12-kDa bands represent different processing intermediates of pro-SPC and that the latter is the more mature form of surfactant protein C (Beers and Mulugeta, 2005). Expression profiles of pro-SPC (Figures 4A–C) show an inverse correlation with ACO1 when queried in the IPF lungs. The expressions of the 12-kDa processing intermediate were less pronounced in the lower versus the upper lobes in IPF lungs (Figure 4A) in contrast to the expression profile of ACO1 which was higher in the lower versus the upper lobes (Figure 2D; Supplementary Figure SE1). Likewise, a significant negative correlation between aconitase activity and the expression of the 12-kDa processing intermediate of pro-SPC was noted (Figure 4B).

### Bleomycin-Induced Lung Fibrosis in Mice Displays an Abundance of Aconitase 1-Positive E-Cadherin-Positive Cells Distributed in Alveolar and Bronchiolar Structures Within Fibrotic Areas

**FIGURE 5:**
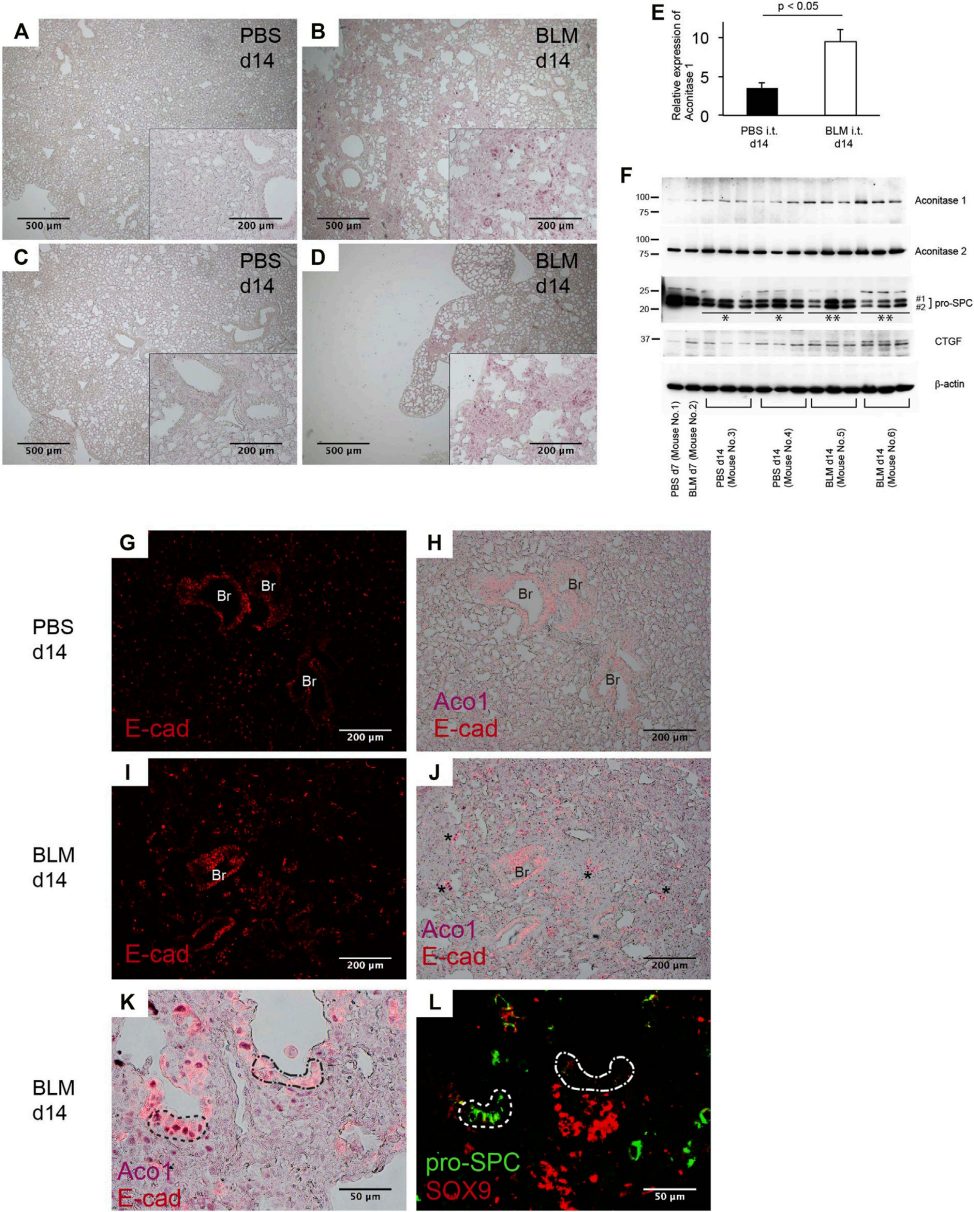
Bleomycin-induced lung fibrosis in mice displays an abundance of aconitase 1-positive E-cadherin-positive cells distributed in alveolar and bronchiolar structures within fibrotic areas. Wild-type mice, aged 7–9 weeks, were intratracheally administered 1.5 U/kg of bleomycin (BLM) or PBS. On days 7 and 14, lung samples were harvested (*n* = 3 each time point/group) **(A–D)** Immunohistochemical (IHC) labeling for ACO1 was performed on paraffin-embedded lung sections from BLM- and PBS-treated mice. The insets at the corner of each photomicrograph are magnified views of each image **(E)** The intensity of ACO1 expression was calculated using IHC images as described in the method section in the online supplement (3 mice for each PBS and BLM group; six or more randomly selected fields at 100X magnification). The results (means ± SE) are shown in bar graphs **(F)** Western blot analysis was performed using whole lysates of the lung tissues. Equal amounts of protein (7.5 μg) were loaded per lane. Each lane grouped within an identical mouse ID (Mouse No. 3, 4, 5, 6) represents an individual sample prepared from different lung lobes **(G–K)** Lung sections were immunohistochemically labeled for E-cad and ACO1. Single-channel images and multi-channel merged images are shown **(L)** Adjacent lung section to K was immunohistochemically labeled for pro-SPC and SOX9. Br: bronchiolar epithelium. A *p*-value of 0.05 or lower was considered to be statistically significant. Data from one representative experiment of two or more independent experiments are shown.

Bleomycin (BLM)-induced pneumopathy in mice is a widely used fibrosis model (Williamson et al., 2015; Tashiro et al., 2017; Carrington et al., 2018). Immunohistochemical staining and western blotting were performed to determine how the expression of ACO1 changes in this model. Quantitative analysis using ACO1-stained lung sections reveals that the intensity of ACO1 signals in BLM-treated lungs is significantly higher than in controls (Figure 5A-E; Supplementary Figure SE4). Western blot data confirm these results (Figure 5F). The expressions of pro-SPC and CTGF (connective tissue growth factor) were evaluated to determine if the highly expressed ACO1 in BLM-treated lungs corresponded to an altered abundance of type II AECs and the associated fibrotic response. The expression of ACO1 exhibited a negative correlation with pro-SPC and a positive correlation with CTGF (Figure 5F). These results indicate that lung fibrosis in BLM-treated lungs is associated with an increase in ACO1 expression. Next, it was determined if the ACO1 signals correlate with VECs in the murine model as occurs in human IPF lungs. Such an association was not found (data not shown). This raises the question as to which cell type(s) express high levels of ACO1 in BLM-treated lungs. Epithelial cells were suspected because strong ACO1 signals in BLM-treated lungs surround the airspace (Supplementary Figure SE4G). Colocalization of ACO1 and E-cad was examined in the lung sections of BLM- or PBS-treated mice by immunostaining to verify this concept. Unlike human IPF lungs, remodeled areas of BLM-treated lungs displayed a wide distribution of cells positive for both E-cad and ACO1 (Figure 5J). Some of these double-positive cells in BLM-treated lungs constitute part of the bronchiole-like structures (asterisks in Figure 5J). Based on these observations, it was postulated that ACO1-positive E-cad-positive cells present in fibrotic areas of BLM-treated lungs signify regeneration of epithelial cells that eventually give rise to type II AECs. Therefore, colocalization of ACO1, E-cad, pro-SPC, and SOX9 [a marker for epithelial cell populations that retain regenerative capacity (Jo et al., 2014; Kang et al., 2016; Nichane et al., 2017)] was investigated by performing immunohistochemical double-labeling on two serially cut sections (Figures 5K, L). The results show that an abundance of cells positive for E-cad, ACO1, and pro-SPC (areas circled by dashed lines in Figures 5K, L) were present in BLM treated lungs. SOX9-positive cell clusters also were observed in the vicinity of these putative regenerating epithelial cells (Figure 5L). This suggests that ACO1 facilitates alveolar regeneration in BLM-induced lung fibrosis in mice.

### Aging Associated With the Patchy Distribution of Aconitase 1-Positive E-Cadherin-Negative Cells in Remodeled Alveoli

**FIGURE 6:**
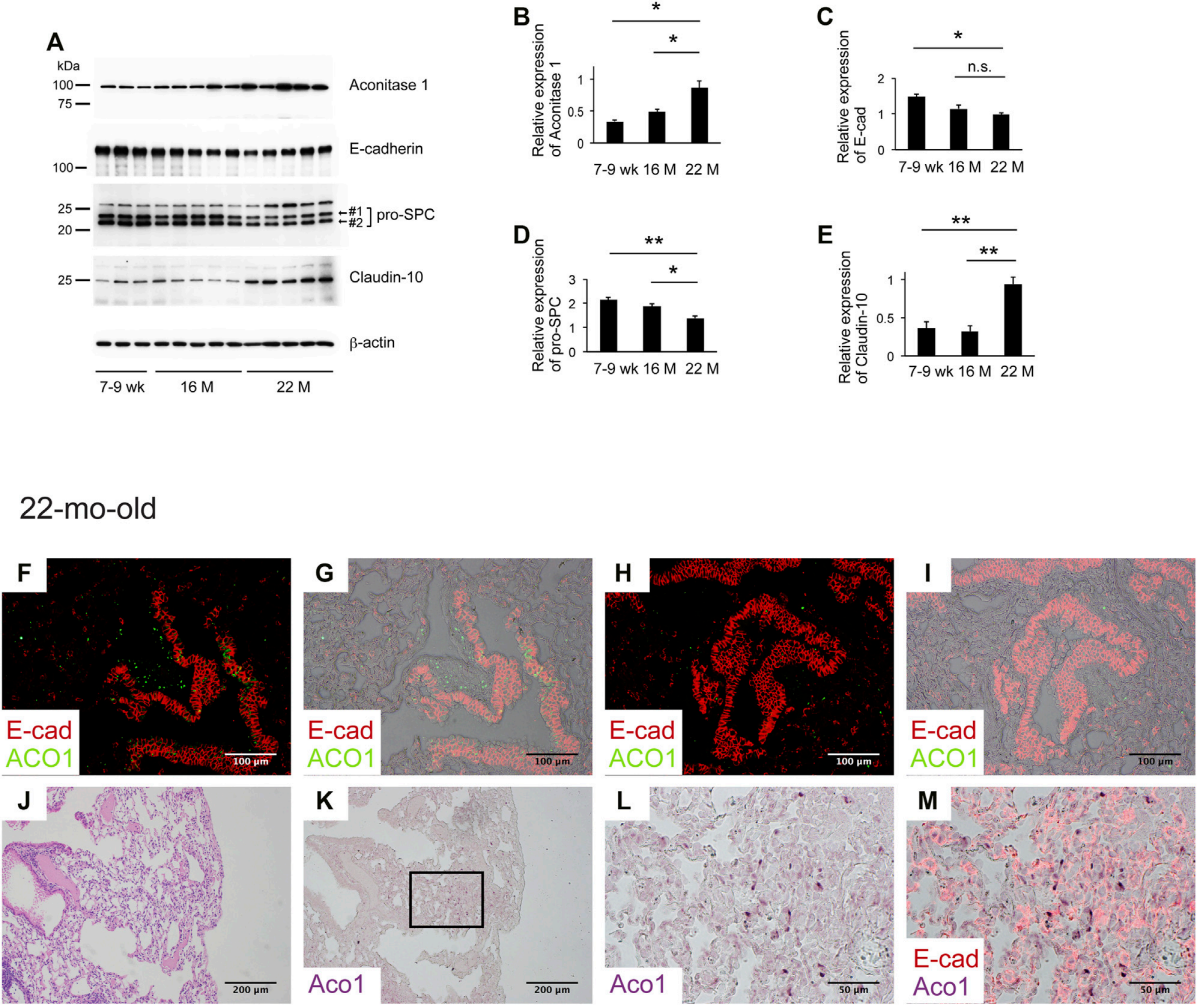
Aging associated with the patchy distribution of aconitase 1-positive E-cadherin-negative cells in remodeled alveoli. **(A)** Western blot analysis was performed using whole tissue lysates prepared from lungs of wild-type mice at different ages [7-9-weeks-old (n = 3), 16-mth-old (n = 5) and 22-mth-old (n = 5)]. Equal amounts of protein (7.5 μg) were loaded per lane. **(B–E)**: Expression of ACO1, E-cadherin, pro-SPC (the sum of #1 and #2 in Figure 6A), and claudin-10 was normalized to β-actin and the results (means ± SE) are shown in bar graphs **(F–M)** Paraffin-embedded lung sections from 22-mth-old wild-type mice were subjected to i) H&E-staining **(J)** or ii) immunohistochemical double labeling for E-cad and ACO1 **(F-I and K-M)**. Single-channel images **(K,L)** and multi-channel merged images **(F-I,M)** are shown. The lung section the photomicrographs of **(K-M)** are imaged from is adjacent to **(J)** **(L, M)** are magnified views of the boxed region in **(K)** *p < 0.05; **p < 0.01. a *p*-value of 0.05 or lower was considered to be statistically significant. Data from one representative experiment of two or more independent experiments are shown.

Aging is associated with decreased lung regeneration (Redente et al., 2011; Thannickal et al., 2014). Studies show that VECs change with age (Kovacs et al., 2014; Mammoto et al., 2019). Data presented above suggest that both ACO1-positive VECs and ACO1-positive lung epithelial cells synergistically contribute to lung regeneration and that i) the expression levels and/or patterns of ACO1 in the lung change with age and ii) such alterations serve as a prerequisite condition for chronic lung diseases. To test the first hypothesis, lungs of 7-9-weeks-, 16-mth- and 22-mth-old mice were collected and western blotting was performed. The results show a significant increase in ACO1 expression in an age-dependent manner (Figures 6A,B).

Next, the expression patterns of ACO1 were characterized. Immunohistochemical double staining for ACO1 and E-cad was performed on lung sections from 7-9-weeks-old and 22-mth-old mice. The results show that the signal intensity of ACO1 in the bronchiolar epithelium of 22-mth-old mice (Figures 6F–I) is comparable to that of 7-9-weeks-old mice (data not shown). However, unlike the lungs of 7-9-weeks-old mice, the 22-mth-old mice displayed patchy ACO1 signals distributed in the interstitium of the bronchovascular bundles (Figures 6F,G) and alveoli (Figure 6F-M). Subsequently, it was determined if ACO1-positive E-cad-positive cells, a putative regenerating epithelial cell population observed in bleomycin-treated lungs, are present in aging lungs. Contrary to expectations, the results show that most of the cells that express ACO1 in aged lungs were negative for E-cad (Figures 6F,H and M). However, these two proteins were frequently detected in close proximity in the subpleural regions (Figure 6M). In addition, microscopic observations indicate the possibility that the intensity of ACO1 signals parallels the degree of loss of normal alveolar structures (Figure 6K). Therefore, it was hypothesized that the increased ACO1 expression in aging lungs reflects emergence of ACO1-positive E-cad-negative epithelial progenitor cells that are unable to differentiate into alveolar epithelial cells.

Therefore, western blotting, using whole lung lysates from mice at different ages, was performed to quantify the expressions of E-cad and pro-SPC, both of which are representative markers that reflect the integrity of type II AECs (Vyas-Read et al., 2014). The results show that E-cad and pro-SPC expression decreased in an age-dependent manner supporting the hypothesis that the age-associated ACO1 increase is a physiological adaptation to cope with the functional loss of type II AECs. Club cells are a progenitor cell type that gives rise to type II AECs (Zemke et al., 2009; Fukumoto et al., 2016). Claudin-10 (cldn10) is a marker for immature club cells and its expression is associated with IPF (Fukumoto et al., 2016). It is hypothesized that age-associated loss of functionally mature type II AECs is addressed physiologically by the emergence of immature club cells committed to type II AECs.

To explore this concept, the expressions of cldn10 in lung lysates of mice at different ages were quantified. Results show that expression of cldn10 increases in an age-dependent manner, supporting the hypothesis that loss of functionally mature type II AECs and emergence of type II AEC-committed club cells is likely age-associated.

## Discussion

The role of ACO1 in IPF has not been previously investigated. For the first time, these data demonstrate that ACO1 expression and aconitase activity correlate with fibrogenesis in IPF. Furthermore, ACO1 expression is localized to the small vessels in the fibrotic areas which suggests that ACO1 may accelerate vascular endothelial cell (VEC) proliferation in IPF (Figure 1F; Supplementary Figure SE2F).

The role of angiogenesis in IPF pathology is controversial; it remains unclear if *de novo* angiogenesis in fibrotic areas of IPF lungs is a compensatory response or plays a causative role in disease progression (Zeinali et al., 2018). This study shows the presence of ACO1-positive proliferative VECs in the fibrotic areas (Supplementary Figures SE3G and, SE3H) versus a paucity of VECs, as demonstrated in a previous study (Ebina et al., 2004). The presence of VECs expressing ACO1 was verified by comparing two serially cut sections from IPF lungs, one stained with H&E and the other immunohistochemically labeled for ACO1 (Supplementary Figure SE2G-L). These results raise the following question: does ACO1 accelerate the proliferation of VECs in fibrotic areas of IPF lungs? Further studies are needed to provide a definitive answer.

Relatively unaffected areas of IPF lungs display a paucity of ACO1 signals in stark contrast to fibrotic lesions with a dense distribution of ACO1-positive small vasculatures (Figure 2E, F). These observations are in contrast to published data showing an increase in CD34-positive capillaries in nonfibrotic lesions of IPF lungs (Ebina et al., 2004). It’s possible that ACO1 is not expressed at high levels in VECs unless they require high metabolism for their migration and proliferation.

Among unexpected findings, ACO1 signals were observed in the nucleus of lung epithelial cells (Figures 1B,C; Figure 5K). ACO1 has a putative nuclear localization signal (NLS) as predicted by the NLS mapper (Supplementary Figure SE5). Accordingly, a 2019 publication revealed that iron regulatory protein 1A (IRP1A), a drosophila ortholog of human ACO1/IRP1, is able to translocate to nuclei (Huynh et al., 2019). This paper indicates that ACO1 enters nuclei in its holo-form, i.e., an iron-sulfur cluster-bound enzymatic form instead of an apo-form or RNA-binding form. The authors surmise that the enzymatic form of ACO1 in the nucleus facilitates an increase in bioavailable iron on an as-needed basis and yet downregulates iron-dependent processes once the iron demand is met. Nuclear ACO1 may help lung epithelial progenitors differentiate into functionally mature type II AECs by finely tuning the intracellular iron availability and thereby facilitating the recruitment of iron-requiring TCA cycle enzymes to the newly generated mitochondria given that ACO1-positive putative regenerating epithelial cells are observed in BLM-treated lungs (Figure 5K, L).

VECs are highly glycolytic. They have metabolic similarities to cancer cells, e.g., high dependence on glycolysis and glutaminolysis for cell growth and proliferation (Metallo et al., 2011; Ratnikov et al., 2015; Wong et al., 2017; Teuwen et al., 2019). Accumulating evidence demonstrates that both VECs and cancer cells reductively carboxylate glutamine-derived α-ketoglutarate (α-KG) to citrate in cytoplasm thereby fueling cell growth and proliferation (Metallo et al., 2011; Mullen et al., 2011; Eales et al., 2016; Hollinshead and Tennant, 2016; Kim et al., 2017; Wong et al., 2017). The activity of this metabolic pathway, which involves ACO1-mediated conversion of isocitrate to citrate, significantly increases when cells are grown under hypoxia (Metallo et al., 2011). It’s plausible that VECs in IPF highly depend on glycolysis and reductive glutaminolysis for their growth and proliferation considering: i) the metabolic similarities between VECs and cancer cells and ii) the hypoxic conditions in which VECs at fibrotic areas of IPF lungs are exposed. This hypothesis is supported by i) the observation that ACO1-positive VECs in fibrotic areas of IPF lungs lack ACO2 expression (data not shown) and ii) literature showing decreased expressions of mitochondrial TCA cycle enzymes including citrate synthase in IPF lungs (Zhao et al., 2017).

The following is a plausible profile of actively proliferating VECs in IPF lungs: i.e. a metabolic state where i) ACO1 actively mediates glutaminolysis and reductive carboxylation in proliferating vascular endothelial cells and ii) only specific linear portions of the mitochondrial TCA cycle actively work to fulfill the anabolic requirements (Mullen et al., 2011; Hollinshead and Tennant, 2016; Huang et al., 2017; Kim et al., 2017; Wong et al., 2017) (Supplementary Figure SE7).

The data presented in this study demonstrate that the specific expression patterns of ACO1 in fibrotic areas of IPF lungs, as manifested by immunohistochemical staining, are not observed in non-IPF chronic lung diseases. Second, the higher expression of ACO1 in the lower lobes of IPF lungs (Figures 2A,C) is associated with more severe fibrosis in the lower lobes as supported by chest computed tomography findings (Supplementary Figure SE6C, D, G, and H).

This study has certain limitations. First, it remains unclear whether high levels of ACO1 in enzymatic form change the behaviors of VECs. Verifying this is difficult since ACO1 appears in two different forms, enzymatic or RNA-binding, depending on the environment. Second, the sample size is limited. Nevertheless, hypothesis-generating observations underlie the foundations for future validation studies. Furthermore, the site-specific heterogeneous expression patterns of ACO1 have been validated by collecting multiple sample blocks from each IPF subject (Figure 2; Figure E1).

**FIGURE 7:**
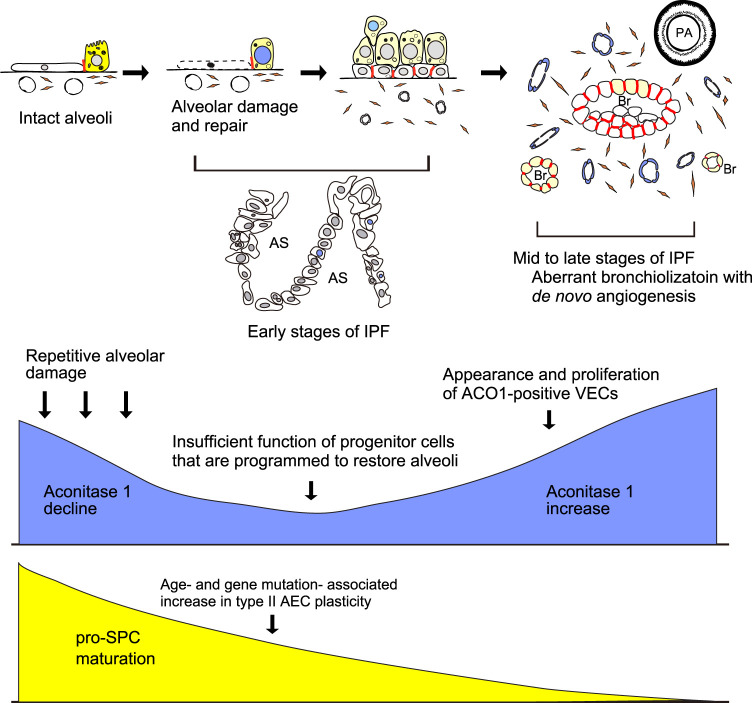
Hypothesized mechanism of how aconitase 1 is involved in IPF development. Aging and genetic predispositions independently increase type II AEC plasticity. In parallel, repetitive injury to alveoli and the associated regeneration process gradually leads to the shortage of type II AEC-committed progenitor cells and accordingly loss of functionally mature type II AECs (early stages of IPF). Low expression of ACO1 in the upper lobes of IPF lungs (Figure 2E, F; Figure E1) corresponds to a paucity of ACO1-positive epithelial cells and the impaired transition from epithelial progenitor to functionally mature type II AECs. Once such disturbed type II AEC regeneration reaches a certain threshold, the conversion of alveoli into bronchioles starts to occur in a random manner. Such aberrant bronchiolization accompanies an uncontrolled *de novo* angiogenesis and fibrosis in the interstitium near the newly formed bronchioles (mid to late phases of IPF with high ACO1 expression). Yellow, blue, and red colors in the schema represent pro-SPC, ACO1, and E-cadherin respectively. AS, airspace; Br, Bronchiole; PA, pulmonary artery; AEC, alveolar epithelial cell; VECs, vascular endothelial cells.

In conclusion, ACO1 expression and aconitase activity parallels the severity of fibrosis in IPF. Furthermore, it is hypothesized that aberrant ACO1 expression in vasculatures is a key event in IPF (Figure 7). These data may improve the understanding of the mechanisms that promote fibrosis in various types of lung diseases.

## Data Availability

The data that supports the findings presented in this study are available on request from the corresponding author.
