# Supplementary material for: Aberrant Expression of ACO1 in Vasculatures Parallels Progression of Idiopathic Pulmonary Fibrosis
Source: Front Pharmacol. 2022 Jul 15;13:890380. doi: 10.3389/fphar.2022.890380 (PMC9335372; doi:10.3389/fphar.2022.890380)
Supplement: Supplementary file 1 [file DataSheet2.PDF]

ONLINE DATA SUPPLEMENT

Aberrant Expression of ACO1 in Vasculatures Parallels Progression of Idiopathic Pulmonary Fibrosis

Jutaro Fukumoto, Muling Lin, Mudassir Meraj Banday, Sahebgowda Sidramagowda Patil, Sudarshan Krishnamurthy, Mason Breitzig, Ramani Soundararajan, Lakshmi Galam, Venkata Ramireddy Narala, Colleen Johns, Kapilkumar Patel, John Dunning, Richard F. Lockey, Nirmal S. Sharma and Narasaiah Kolliputi

DETAILED MATERIALS AND METHODS

Cell culture

A549 and H441 cells were cultured in RPMI1640 medium supplemented with 10% fetal bovine serum (FBS) and 1% penicillin-streptomycin (P/S). Primary lung fibroblasts isolated from lungs of mice were cultured in DMEM medium supplemented with 10% FBS and 1% penicillin-streptomycin.

Human Pulmonary Bronchial Epithelial Cells (PBEC; cat# PCS-300-010, ATCC, Manassas, VA) and Human Lung Microvascular Endothelial Cells (HMVEC; cat# CC-2527, Lonza, Walkersville, MD) were maintained in BEGM Bronchial Epithelial Cell Growth Kit (Cat# PCS-300-040, ATCC) and EGM-2 MV bullet kit (Cat# CC-3202, Lonza), respectively. Both PBECs and HMVECs were cultured in a humidified 5% CO2 incubator at 37°C.

T7 cells, mouse type II AECs, (cat# 07021402, Sigma-Aldrich, St. Louis, MO), and C22 cells, a mouse club cell line (cat# 07021401, Sigma-Aldrich), were maintained in DMEM media containing 0.25 μg/ml human endothelin-1 (cat# 331-25, California Peptide Research, Inc.), 0.01 μg/ml human interferon gamma (IFN-γ; cat# 300-02, PeproTech), 10 μg/ml bovine insulin (cat# 700-112P, Gemini Bio-Products), 5 μg/ml human transferrin (cat# 16-16-032001-LEL, Athens Research & Technology), 7.5 μg/ml bovine Endothelial Cell Growth Supplement (cat# 356006, Corning), 0.025 μg/ml mouse epithelial growth factor (cat# PMG8041, Thermo Fisher Scientific, Waltham, MA), 0.36 μg/ml hydrocortisone (cat# 354203, Thermo Fisher Scientific), 0.02 μg/ml 3,3',5-Triiodo-L-thyronine (cat# T2877, Sigma-Aldrich). Both T7 and C22 cells are cultured in a humidified 8-10% CO2 incubator at 37°C.

Isolation of mouse lung fibroblasts

Primary fibroblasts were isolated from lungs of wild-type (WT) mice at 7-9 wks of age using enzymatic digestion. Briefly, isolated mouse lungs were minced and sequentially digested for 30 min at 37℃ in DMEM medium 3 mg/ml of collagenase and 125 U/ml hyaluronidase. A sufficient amount of DMEM media containing 10% FBS 1% P/S was added to digested tissues. The whole cell suspension was transferred to 100 mm cell culture dishes and grown for 48-72 h under hypoxic conditions with 3% oxygen/5% carbon dioxide using proOx C21 and C-Chamber (BioSpherix, NY, USA). Cells were returned to 20% oxygen/5% carbon dioxide, and the medium was replaced every day until the cells floating in the media were removed. Cells were trypsinized and spread at 80-90% confluence. Cells between passage 2 and 3 were harvested for protein extraction.

Plasmids and transfection

The medium was replaced with serum-free Opti-MEM I medium (cat# 51985034; Thermo Fisher Scientific) when the density of A549 or H441 cells reached 70–80% confluence. Subsequently, for the control group, cells were transfected with one of the following empty vectors using Lipofectamine 2000 (cat# 11668019; Invitrogen, Life Technologies, Carlsbad, CA): pCMV6-Entry (OriGene Technologies, Rockville, MD) or pcDNA3.1/V5-His-TOPO (a gift from Dr. Pascale Fanen; Mondor Institute of Biomedical Research, Paris, France). For the overexpression group, either, pCMV6-Entry-Aconitae 1 (OriGene Technologies) or pcDNA3.1/V5-His-TOPO-pro-SPC^{WT} (a gift from Dr. Pascale Fanen) was transfected. Three hours after transfection, the Opti-MEM I medium containing plasmid DNA and transfection reagent was replaced with complete medium. Twenty-four hours after transfection, cells were harvested and whole cell

lysates were prepared. Primary fibroblasts isolated from lungs from healthy patients and those with IPF (cat# CC-2512; Lonza, Basel, Switzerland) were seeded on cell culture plates in DMEM medium (cat. No. 15-013-CV; Cellgro, Herndon, VA) supplemented with 10% FBS, 2 mM glutamine, 1 unit/mL penicillin-streptomycin and 2.5 ng/mL Amphotericin B. Cells were spread when their density reached 70–80% confluence. Cells at passages 3–4 were harvested for preparation of protein lysates.

Mice

All procedures were approved by the Institutional Animal Care and Use Committee of the University of South Florida. C57BL/6J mice (Harlan laboratories, Indianapolis, IN or Envigo, Indianapolis, IN) at 6–7 wks of age were purchased and housed in individually ventilated cage/rack systems until they reached the ages designated for each experiment (7-9-wk-, 16-month-, and 22-month-old). Mice had ad libitum access to food and water.

Bleomycin administration on mice

Mice aged 7–9 wk, age- and sex-matched, were randomly assigned to either the control group or the treatment group. After anesthetizing the mouse, the skin of the anterior cervical region was cut. Subsequently, part of the tracheal sheath was removed, using a pair of fine point tweezers, to expose the anterior wall of the trachea. 50 µl of sterile PBS (control group) or bleomycin (1.5 U/kg; treatment group) dissolved in 50 µl of PBS was injected into the lung through the tracheal lumen using a 1 ml syringe with a needle. The skin incision was closed using a polypropylene monofilament suture.

Collection of mouse lung

Mouse lung samples were collected as described previously (1). Briefly, mice were euthanized by intraperitoneal injection of a ketamine/xylazine mixture followed by cervical dislocation. Following thoracotomy, the lungs were perfused using phosphate-buffered saline (PBS). For histological analysis, either the whole lung or left lobe was fixed in 10% neutral buffered formalin for 48-72 h. The sample was subsequently embedded in paraffin. For other purposes, the lung samples were snap-frozen and stored in liquid nitrogen until use.

Aconitase activity assay

Sample preparation for the aconitase activity assays was conducted as follows: Frozen lung tissue samples that had been stored in -80°C, were pulverized using tissue pulverizers (cat# 08-418-2, Thermo Fisher Scientific). Subsequently, pulverized lung samples were collected in 1.5 or 2.0 mL centrifuge tubes and stored at -80°C until use. One or more days before the activity assay, part of the pulverized lung samples was transferred to a new tube and resuspended in homogenization buffer (50 mM Tris, 0.2 mM sodium citrate, pH 7.4) on ice, then homogenized with 60 strokes using the Biomasher II tissue grinder (Kimble Chase, Vineland, NJ). The lung homogenates were immediately snap-frozen in liquid nitrogen and transferred to a -80°C freezer. After storage at -80°C for more than a day, the samples were thawed and centrifuged at 15,000 g for 10 min at 4°C. The supernatants were taken as samples for the aconitase activity assays. The activity of aconitase in the lung homogenate was measured using a commercially available enzymatic assay kit (cat# MAK051, Sigma-Aldrich) following the manufacturer’s instructions, with some modifications. Each sample used in the aconitase activity assay was also subjected to a BCA assay (cat# 23225, Thermo Fisher Scientific) to determine protein concentration and

normalize activity to the protein concentration (Figure 3 & 4).

Human lung samples

All lung samples used in the current study were excised from lung explants of patients undergoing lung transplantation at Tampa General Hospital (IRB protocol Pro00032158) for various lung diseases: IPF (n=9), chronic obstructive pulmonary disease (COPD; n=1), pulmonary arterial hypertension (PAH; n=1), cystic fibrosis (CF: n=1), dermatomyositis-associated interstitial lung disease (DM-ILD; n=1), mixed connective tissue disease (MCTD: n=1) and chronic hypersensitivity pneumonitis (HP; n=2) following lung transplant. Sample cutting was performed in the same operation room as transplantation, and was started less than one hour after the native lungs were removed from the recipient. From the native lungs of each recipient, 6 lung blocks were collected: 3 from either the right or left upper lobe and 3 from either the right or left lower lobe, with some exceptions [Patient No.1 (6 samples from right lower lobe); Patient No.4 (3 samples from right lower and 3 samples from left lower lobes); Patient No.9 (6 samples from left lower lobes)]. We ensured that samples were collected from the subpleural region of each lobe and approximately two inches from the pleura. Each block was split in two parts in such a way that the cutting plane was perpendicular to the pleura. One part was frozen at -80°C for multiple purposes including protein analysis, and the other immersed in 10% neutral buffered formalin for histological study.

Routine and immunohistochemical staining on lung tissue sections

Routine morphological evaluation was performed using lung tissue sections stained with

hematoxylin and eosin (H&E). Immunohistochemical (IHC) staining was performed on paraffin-embedded lung tissue sections. Briefly, paraffin sections were deparaffinized in xylene. After being washed in ethanol gradients and PBS, sections were subjected to heat-induced antigen retrieval (HIAR) in Tris buffer (10 mM Tris, 1 mM EDTA, 0.05% Tween 20, pH 9.0). Following HIAR, sections were incubated in 3% hydrogen peroxide and PBS for 20 min to block endogenous peroxidase activity, and then incubated with PBS containing 5% BSA and 0.25% Tween-20 for an additional 20 min. Sections were then incubated with primary antibodies and subsequently fluorescence-labeled or HRP-conjugated secondary antibodies. When the sections needed to be labeled for two or more different proteins, this antibody incubating process was repeated separately. Detection of the HRP-conjugated antibodies was performed using ImmPACT VIP Peroxidase (HRP) Substrate (Vector Laboratories, Burlingame, CA). Digital images were taken using a microscope (Olympus BX43; Olympus, Tokyo, Japan) connected to an Olympus DP21 digital camera.

Antibodies

The following antibodies were used: rabbit anti-Aconitase 1 antibody (cat# PA5-41753, Thermo Fisher Scientific), rabbit anti-Aconitase 2 antibody (cat# 6922S, Cell Signaling, Danvers, MA), horseradish peroxidase (HRP)-conjugated rabbit anti-β-actin antibody (cat# 5125S, Cell Signaling), rabbit anti-prosurfactant protein C (pro-SPC) antibody (cat# AB3786, MilliporeSigma, St. Louis, MO; used for western blotting), rabbit anti-pro-SPC antibody (cat# WRAB-9337, Seven Hills Bioreagents, Cincinnati, OH; used for immunostaining), rabbit anti-connective tissue growth factor (CTGF) antibody (cat# ab6992, Abcam, Cambridge, MA), mouse anti-Ki67 antibody (cat# 9449S, Cell Signaling, Danvers, MA), rabbit anti-Ki67 antibody

(cat# AB9260, MilliporeSigma), goat-anti-SOX9 antibody (cat# AF3075, R&D Systems, Minneapolis, MN), rabbit anti-SOX9 antibody (cat# ab185230, Abcam), goat anti E‒cadherin antibody (cat# AF748, R&D Systems), rabbit anti-claudin-10 antibody (cat# 38-8400, Life Technologies, Carlsbad, CA), Alexa Fluor 488-conjugated donkey anti-rabbit IgG antibody (cat# 711-546-152, Jackson ImmunoResearch Laboratories, Bar Harbor, ME), Alexa Fluor 594-conjugated donkey anti-goat IgG antibody (cat# 715-586-150, Jackson ImmunoResearch Laboratories), Alexa Fluor 594-conjugated donkey anti-mouse IgG antibody (cat# 715-586-150, Jackson ImmunoResearch Laboratories), HRP-conjugated donkey anti-rabbit IgG antibody (cat# A16035, Thermo Fisher Scientific), HRP-conjugated donkey anti-mouse IgG antibody (A16017, Thermo Fisher Scientific), and HRP-conjugated donkey anti-goat IgG antibody (cat# A16005, Thermo Fisher Scientific). Detection of the HRP-conjugated antibodies was performed using ImmPACT VIP Peroxidase (HRP) Substrate (cat# SK4605, Vector Laboratories, Burlingame, CA).

Quantification of protein expression for immunohistochemically stained lung section

Quantification of the gross signal intensity for the targeted protein in each of the immunohistochemically labeled sections was performed using ImageJ ver 2.0.0/FIJI referring to a previous paper (2). Briefly, the original RGB images were split into three channels using the plugin “Colour Deconvolution” based on the parameters that had been optimized to exclusively extract the protein signals. The threshold was set to one of the three channels that reflected the targeted signals. Images were converted to black and white (Figure E4), and then the gross signal intensity of the targeted protein in each image was calculated using the software.

Western blotting

Protein lysates were prepared as follows. For tissue samples, frozen lung tissue that had been stored at -80°C or liquid nitrogen temperature, was pulverized. Subsequently, pulverized lung samples were homogenized in lysis buffer [20 mM Tris HCl (pH 7.4), 150 mM NaCl, 0.5% Triton X-100] supplemented with protease and phosphatase inhibitors (Sigma-Aldrich, cat# P8340 and P0044) using a Biomasher II tissue grinder (Kimble Chase, Vineland, NJ). Crude homogenates of the lung were sonicated and sequentially centrifuged at 15,000 g for 10 min at 4°C. The supernatants were taken as whole lysates of the lung for western blot analysis. Regarding cells, cultured cells were washed using ice-cold PBS, collected in centrifuge tubes, and spun down. After removal of the supernatant, the cell pellets were resuspended with RIPA buffer (Thermo Fisher Scientific) containing protease and phosphatase inhibitors. Samples were further sonicated and used as protein lysates for western blotting. Protein samples were mixed with 4x SDS sample buffer (Boston BioProducts, Worcester, MA) and boiled for 5 min at 95°C. Equal amounts of protein were subjected to sodium dodecyl sulfate polyacrylamide gel electrophoresis (SDS-PAGE). Proteins were then transferred onto polyvinylidene difluoride (Thermo Fisher Scientific) or nitrocellulose membranes (GE Healthcare, Waukesha, WI). The membranes were blocked in Tris-buffered saline (20 mM Tris·HCl at pH 7.5 and 150 mM NaCl) with 0.1% Tween 20 (TBS-T) containing 5% skim milk, and then incubated with primary antibodies overnight. The membranes were then washed with TBS-T and incubated with HRP-conjugated secondary antibody (Jackson ImmunoResearch Laboratory, Inc., West Grove, PA) for 60 min at room temperature. The proteins were visualized using Pierce ECL western blotting substrate (Thermo Fisher Scientific) or SuperSignal West Femto maximum sensitivity substrate (Thermo Fisher Scientific). Detection of the protein bands were performed on autoradiography

film (MIDSCI #BX57, St. Louis, MO) or using a Kwik Quant Imager (Kindle Biosciences, LLC).

Prediction of nuclear localization signal sequences

The domain diagram in this paper was prepared with chimera, a visualization system for exploratory research and analysis (3) using the structure of human ACO1/IRP1, PDB-ID 2B3X available at protein data bank (4). The prediction of nuclear localization signal sequences (NLS) was performed using NLS mapper (http://nls-mapper.iab.keio.ac.jp/cgi-bin/NLS_Mapper_form.cgi) (5). The predicted NLS for ACO1 which showed a highest score was used to make 2D and 3D domain structures for ACO1 (Figure E5).

Statistical analysis

For parametric and non-parametric two-group comparisons Student's t-test and Mann-Whitney U-test were used respectively. When multiple comparisons were performed on data comprised of two or more groups, the p-value was adjusted using Bonferroni's method.

REFERECES

E1. Fukumoto J, Fukumoto I, Parthasarathy PT, Cox R, Huynh B, Ramanathan GK, Venugopal RB, Allen-Gipson DS, Lockey RF, Kolliputi N. NLRP3 deletion protects from hyperoxia-induced acute lung injury. *Am J Physiol Cell Physiol* 2013; 305: C182-189.

E2. Rizzardi AE, Johnson AT, Vogel RI, Pambuccian SE, Henriksen J, Skubitz AP, Metzger GJ, Schmechel SC. Quantitative comparison of immunohistochemical staining measured by digital image analysis versus pathologist visual scoring. *Diagn Pathol* 2012; 7: 42.

E3. Pettersen EF, Goddard TD, Huang CC, Couch GS, Greenblatt DM, Meng EC, Ferrin TE. UCSF Chimera--a visualization system for exploratory research and analysis. *J Comput Chem* 2004; 25: 1605-1612.

E4. Dupuy J, Volbeda A, Carpentier P, Darnault C, Moulis JM, Fontecilla-Camps JC. Crystal structure of human iron regulatory protein 1 as cytosolic aconitase. *Structure* 2006; 14: 129-139.

E5. Kosugi S, Hasebe M, Tomita M, Yanagawa H. Systematic identification of cell cycle-dependent yeast nucleocytoplasmic shuttling proteins by prediction of composite motifs. *Proc Natl Acad Sci U S A* 2009; 106: 10171-10176.

E6. Zhao YD, Yin L, Archer S, Lu C, Zhao G, Yao Y, Wu L, Hsin M, Waddell TK, Keshavjee S, Granton J, de Perrot M. Metabolic heterogeneity of idiopathic pulmonary fibrosis: a metabolomic study. *BMJ Open Respir Res* 2017; 4: e000183.

Figure E1

The expression of ACO1 is high in the lower lobes of IPF lungs, versus the upper lobes

(A-G) Western blot analysis for Aconitase 1 (ACO1) was performed using whole lysates prepared from the native lungs of the recipients of lung transplantation performed against varied lung diseases. Each lane represents individual sample from different sampling site (please see Figure 3A). Equal amounts of protein (7.5 g) were loaded per lane. COPD (chronic obstructive pulmonary disease); PAH (pulmonary arterial hypertension); CF (cystic fibrosis); DM-ILD (dermatomyositis-associated interstitial lung disease); HP (chronic hypersensitivity pneumonitis); MCTD (mixed connective tissue disease); HP (chronic hypersensitivity pneumonitis); IPF (idiopathic pulmonary fibrosis). (H-N) Expression of ACO1 in western blot results was normalized to β-actin, and the relative ACO1 expression for each of the sampling site was expressed in bar graphs. The data from patients with IPF (Patient No. 6-10, 13, 14) are shown. Data from one representative experiment of two or more independent experiments are shown.

Figure E2

High levels of Aconitase 1 expression in the interstitium of fibrotic areas of IPF lungs

(A-F) Paraffin-embedded lung sections from patients with varied lung diseases were subjected to H&E-stain (G, H, J, K), or immunohistochemically labeled for i) Aco1 (A-C, I, L) or ii) E-cad, ACO1 and vWF (D-F). The inset in the lower right corner is a magnified view for each panel (A, B, C). Br = bronchiolar lumen. (A) Normal-looking alveoli display widely distributed ACO1 signals. ACO1 signals are noted in both airspace (areas circled by dashed lines) and interstitium (arrows). (B) Highly remodeled area with no airspace left displays a paucity of ACO1 signals.

(C) Highly remodeled area from subpleural region exhibits an abundance of ACO1 signals that largely overlapped with tubular structures (asterisiks). (D) Alveolar area with slightly thickened interstitium exhibits ACO1 signals in both airspace and interstitium. vWF signals highlight the endothelium of several arteries (asterisks). (E) Area that contains highly remodeled lesion and normal looking alveoli. The former displays co-localization of ACO1 and vWF signals. (F) Highly remodeled area with remarkable bronchiolization exhibits an abundance of ACO signals that scatter in the interstitium as well as in the artery. (G-L) The lung section that **G** and **H** were imaged from is adjacent to the one that **I** was imaged from. Likewise, the lung section that **J** and **K** were imaged from is adjacent to the ones that **L** was imaged from. (G-I) Highly remodeled area featuring massive infiltration of inflammatory cells show vascular endothelial cells expressing high levels of Aconitase 1 (areas circled by dot-dashed lines). Aberrant deposition of collagen fibers and abundance of hemosiderin-laden macrophages are noted in the interstitium near the vasculatures. (J-L) Highly remodeled area that shows aberrant bronchiolization is shown. In contrast to the bronchiolar epithelium that weakly express ACO1 (areas circled by dashed lines), the vascular structures that surround the bronchioles express high levels of ACO1 (areas circled by dot-dashed lines). Data from one representative experiment of two or more independent experiments are shown.

Figure E3

ACO1-positive cells are proliferating near bronchioles in highly remodeled area in IPF lungs.

(A-F) Paraffin-embedded lung sections from patients with varied lung diseases were immunohistochemically triple labeled for ACO1, E-cadherin and Ki67. Merged images from

patient with COPD (chronic obstructive pulmonary disease, **A**), MCTD (mixed connective tissue disease, **B**) and IPF (**C**-**F**), are shown. (G and H) Paraffin-embedded lung sections from patients with IPF were immunohistochemically triple labeled for pro-SPC, E-cadherin and Ki67. The inset in the lower right corner is a magnified view for each panel. (**D**, **E**, **F**) are magnified views of the boxed regions in **C**. **H** is a magnified view of the boxed region in **G**. Data from one representative experiment of two or more independent experiments are shown.

Figure E4

Conversion of RGB image obtained by immunohistochemical labeling to black and white image.

(A, C, E and G) The original RGB images obtained by immunohistochemical labeling for Aconitase 1 are shown. (B, D, F and H) The original RBG images were converted to black and white images for quantitative analysis using ImageJ ver 2.0.0/FIJI (see details for method section in the manuscript).

Figure E5

Predicted location of nuclear localization signal (NLS) for Aconitase 1

2D and 3D Domain structures for Aconitase 1 (ACO1) and the location of the predicted nuclear localization signal (NLS) are shown.

Figure E6

High-resolution computed tomography (HRCT) of the lung for the patients whose lung samples were used in the current study.

Each patient listed on the Table 1 received high-resolution computed tomography (HRCT) of the lung as part of his or her pre-transplantation work-up. These images demonstrate the severity of patient's lung disease prior to transplantation. The CT images shown here are from Patients No, 2 (PAH), No. 5 (HP), No. 7 (IPF), and No. 10 (IPF). The yellow dashed lines designate the lobes from which lung specimens were collected (please refer to Table 1). LU: left upper, LL: left lower, RU: right upper, RL: right lower.

Figure E7

Hypothesized metabolic state of actively proliferating vascular endothelial cells in IPF lungs.

Enzymes and molecules that are shown in red were reported to be upregulated or high in amount in IPF vs non-IPF controls while those in blue were reported to downregulated or low in amount in IPF [please see reference (6) for details]. Thicker lines represent actively proceeding pathways.
