# Supplementary figures and images for: Aberrant Expression of ACO1 in Vasculatures Parallels Progression of Idiopathic Pulmonary Fibrosis
Source: Front Pharmacol. 2022 Jul 15;13:890380. doi: 10.3389/fphar.2022.890380 (PMC9335372; doi:10.3389/fphar.2022.890380)

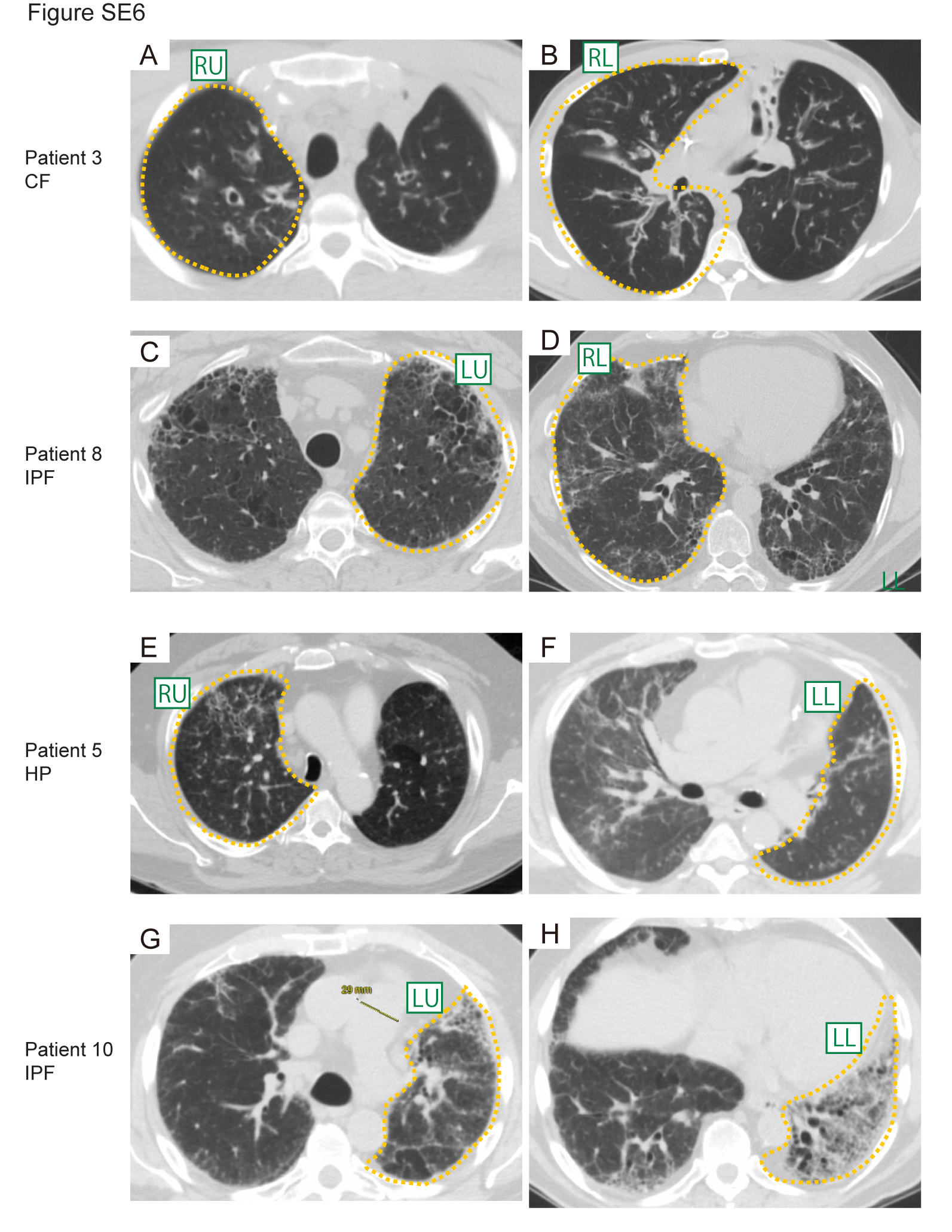

Supplement: Supplementary file 2 [file Image6.tif]

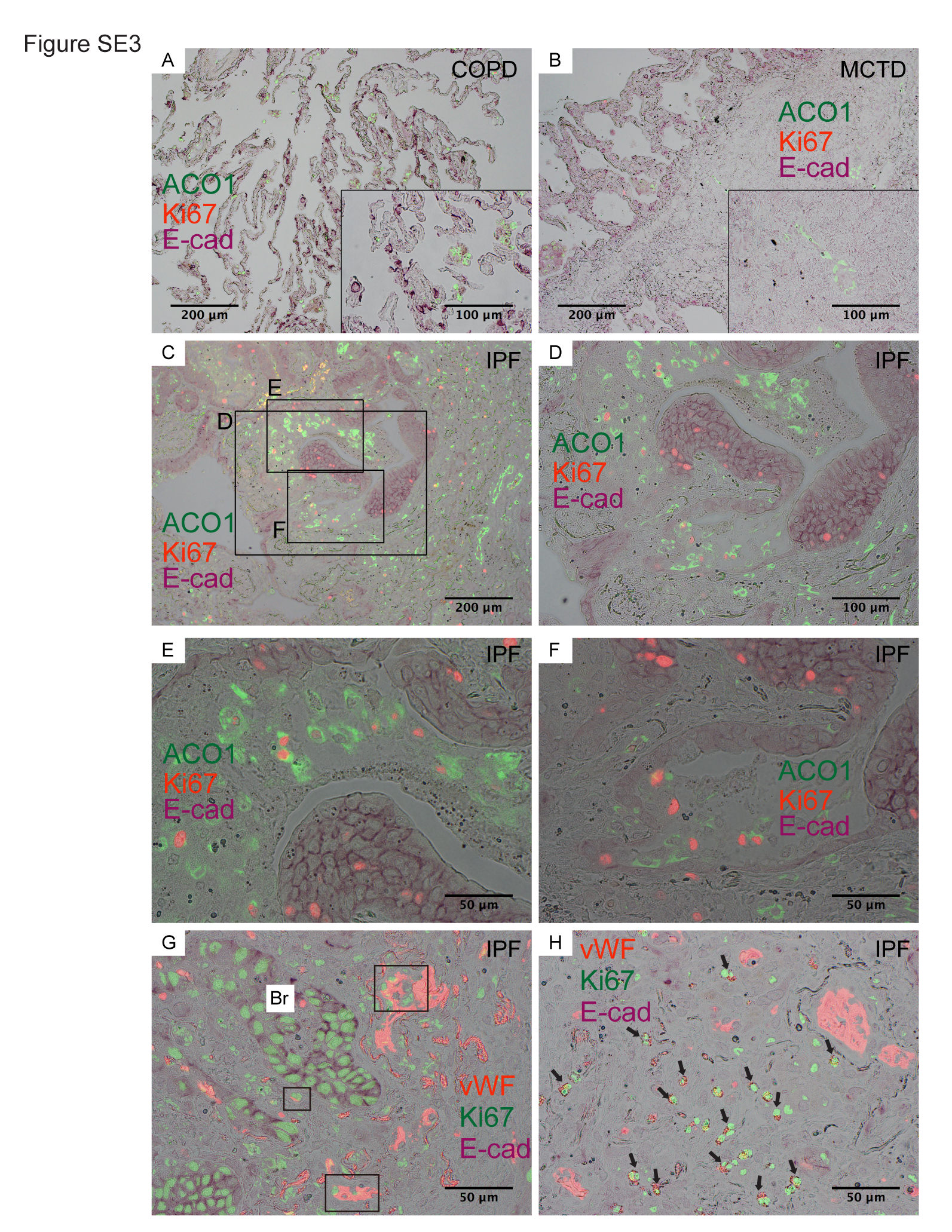

Supplement: Supplementary file 3 [file Image3.tif]

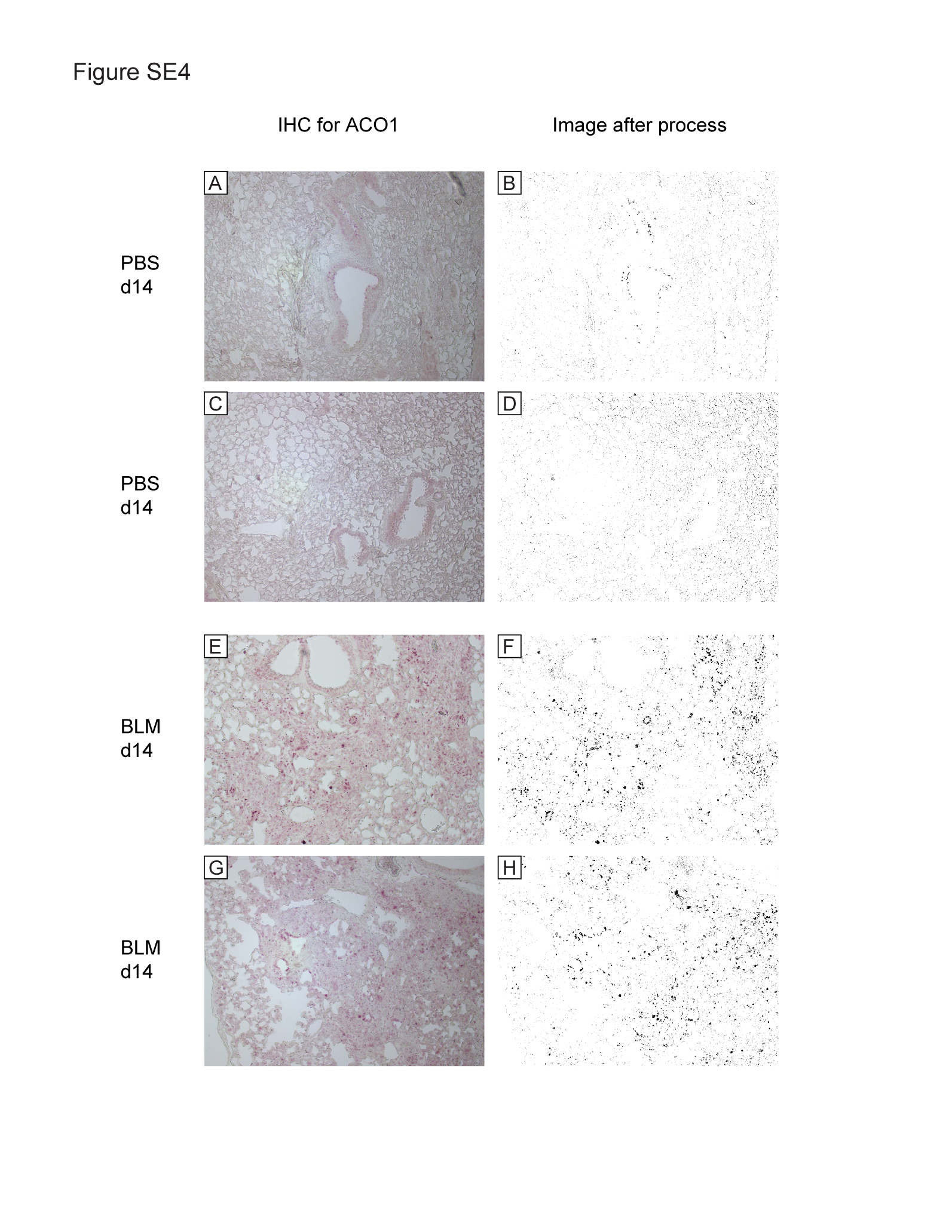

Supplement: Supplementary file 4 [file Image4.tif]

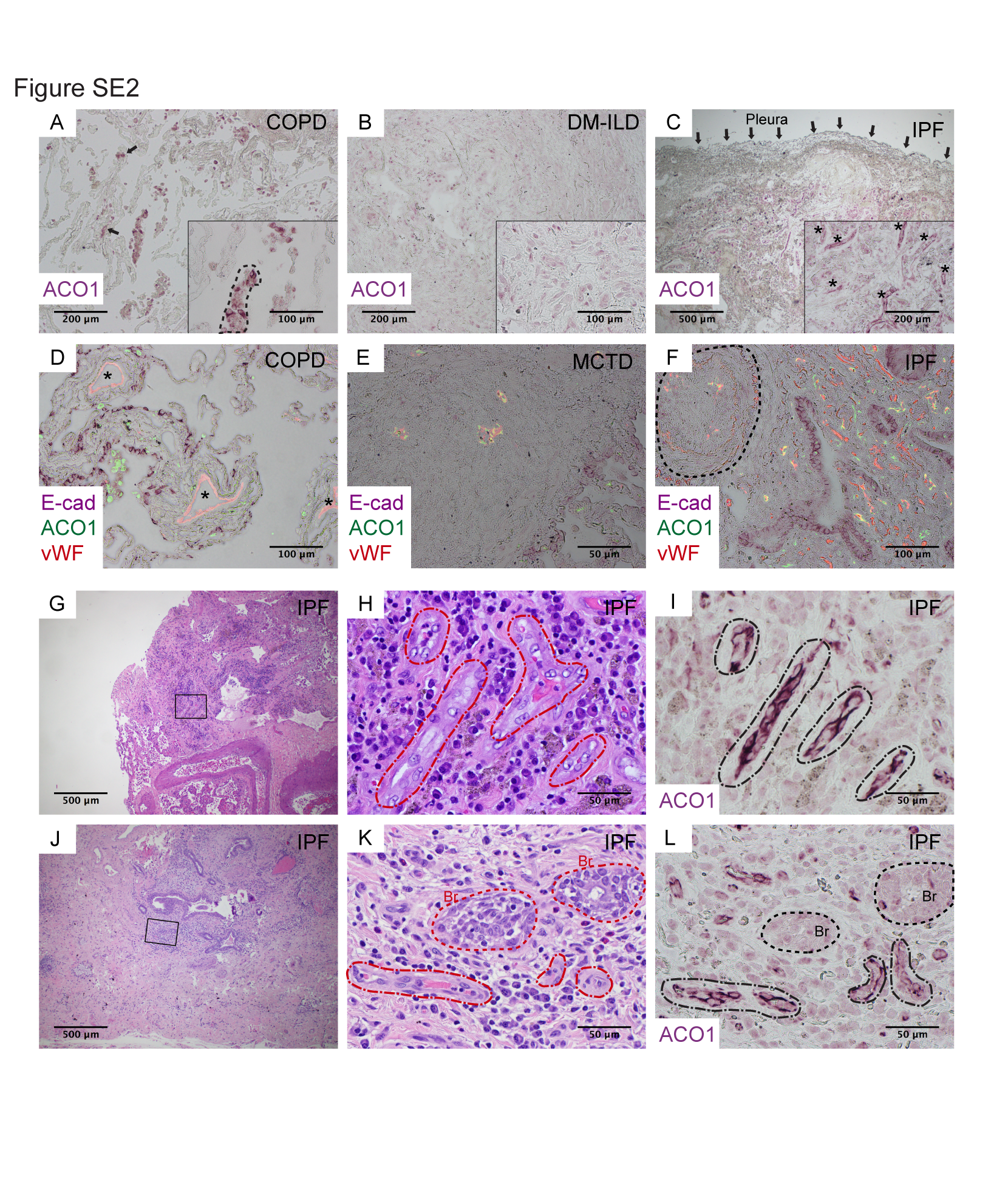

Supplement: Supplementary file 5 [file Image2.tif]

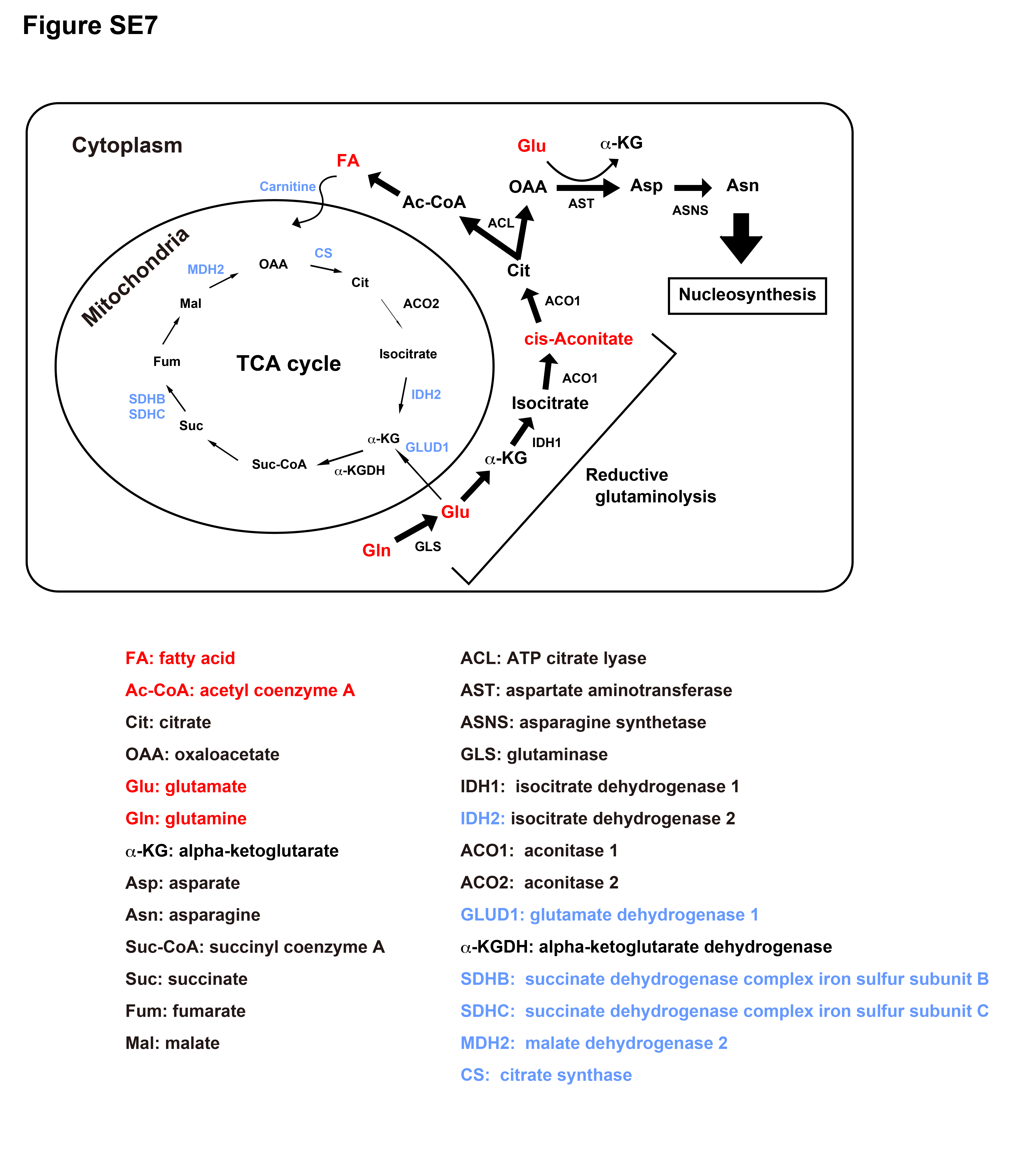

Supplement: Supplementary file 7 [file Image7.jpg]

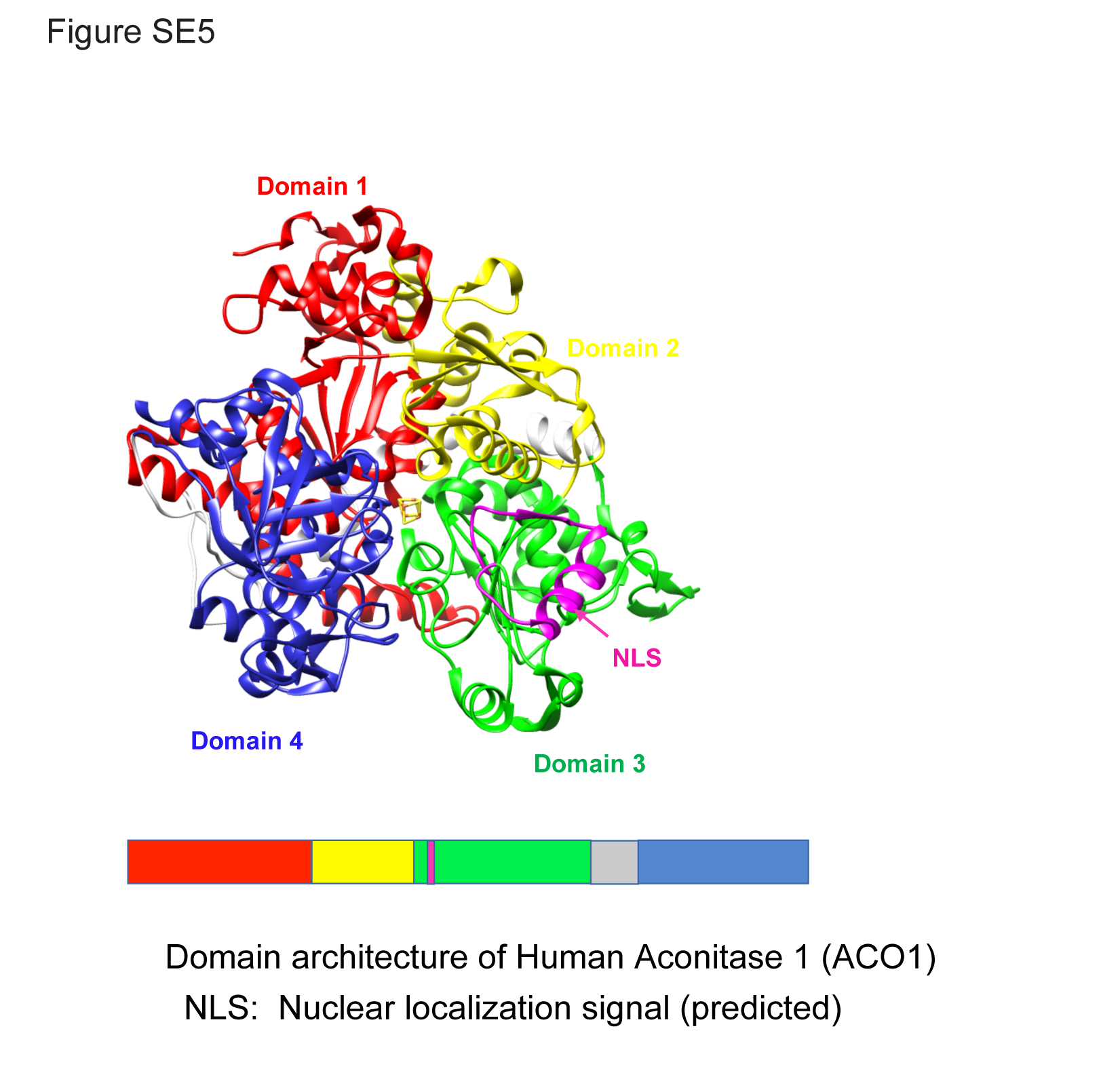

Supplement: Supplementary file 8 [file Image5.tif]

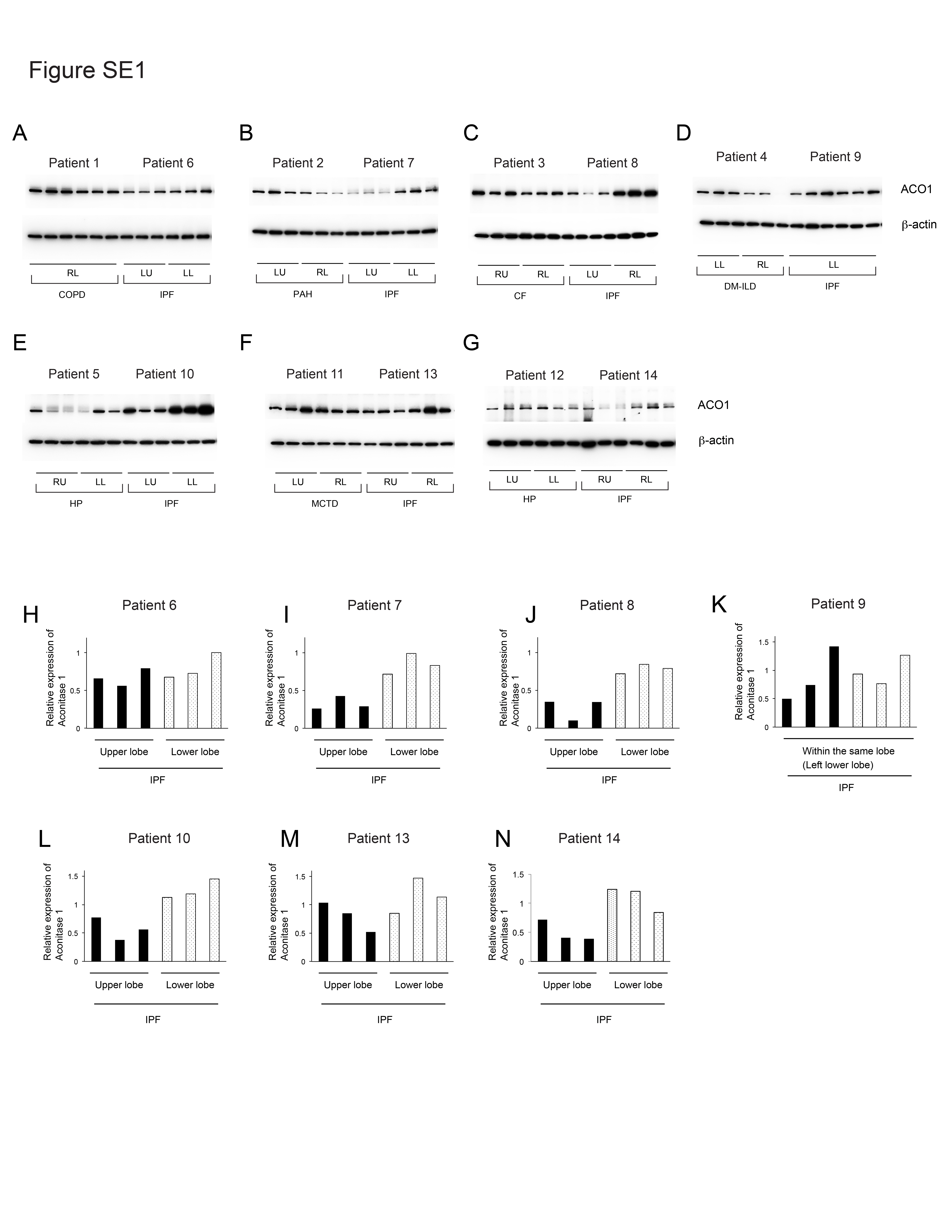

Supplement: Supplementary file 9 [file Image1.jpg]
