# Supplementary material for: Aberrant Expression of ACO1 in Vasculatures Parallels Progression of Idiopathic Pulmonary Fibrosis
Source: Front Pharmacol. 2022 Jul 15;13:890380. doi: 10.3389/fphar.2022.890380 (PMC9335372; doi:10.3389/fphar.2022.890380)
Supplement: Supplementary file 6 [file DataSheet1.PDF]

Table 1

Pt No	Age	Gender	Race	Dx	FVC	FEV1
1	67	M	White	COPD	3.18 (68)	1.11 (31)
2	41	M	White	PAH	3.73 (57)	2.66 (52)
3	30	M	White	CF	2.17 (44)	0.97 (24)
4	46	M	Hispanic	DM-ILD	3.60 (59)	2.62 (63)
5	55	M	White	Chronic HP	1.66 (41)	1.37 (43)
6	58	F	Black	IPF	1.11 (47)	0.76 (40)
7	69	M	White	IPF	2.08 (52)	1.92 (62)
8	47	M	White	IPF	3.57 (65)	2.74 (63)
9	61	M	White	IPF	1.33(34)	1.01 (32)
10	67	M	White	IPF	2.07 (47)	1.62 (47)
11	61	F	Black	MCTD	1.64 (69)	1.02 (53)
12	61	M	White	Chronic HP	1.30 (27)	1.16 (38)
13	65	M	White	IPF	1.54 (33)	1.32 (36)
14	67	M	White	IPF	3.10 (66)	2.10 (60)
15	65	F	White	IPF	0.85 (31)	0.75 (35)
16	52	M	White	IPF	1.47 (34)	1.20 (34)
